# Orthogonal regulation of DNA nanostructure self-assembly and disassembly using antibodies

**DOI:** 10.1038/s41467-019-13104-6

**Published:** 2019-12-03

**Authors:** Simona Ranallo, Daniela Sorrentino, Francesco Ricci

**Affiliations:** Chemistry Department, University of Rome, Tor Vergata, Via della Ricerca Scientifica, 00133 Rome, Italy

**Keywords:** Supramolecular chemistry, DNA nanotechnology, Nanobiotechnology

## Abstract

Here we report a rational strategy to orthogonally control assembly and disassembly of DNA-based nanostructures using specific IgG antibodies as molecular inputs. We first demonstrate that the binding of a specific antibody to a pair of antigen-conjugated split DNA input-strands induces their co-localization and reconstitution into a functional unit that is able to initiate a toehold strand displacement reaction. The effect is rapid and specific and can be extended to different antibodies with the expedient of changing the recognition elements attached to the two split DNA input-strands. Such an antibody-regulated DNA-based circuit has then been employed to control the assembly and disassembly of DNA tubular structures using specific antibodies as inputs. For example, we demonstrate that we can induce self-assembly and disassembly of two distinct DNA tubular structures by using DNA circuits controlled by two different IgG antibodies (anti-Dig and anti-DNP antibodies) in the same solution in an orthogonal way.

## Abstract (web summary)

Antibodies are useful biomarkers and are emerging as powerful therapeutic tools. Here the authors report a rational strategy to orthogonally control assembly and disassembly of DNA-based nanostructures using specific IgG antibodies as molecular inputs.

## Introduction

Antibodies (immunoglobulins) are proteins produced by the immune system in response to foreign compounds and infectious agents. Because of their biological role antibodies are among the most important disease biomarkers and their level in clinical samples is routinely used to diagnose a wide range of pathologies including infectious and auto-immuno diseases^1,2^. The use of antibodies is also gaining importance in therapeutic settings with immunotherapy rapidly becoming one of the most promising treatment strategies in oncology^3–5^. Given this crucial role, it becomes mandatory to create new nanoscale biotechnological tools that can respond to the presence of specific antibodies and could be used for fundamental research and clinical applications (drug-delivery and diagnostic). Despite this, however, only few examples have demonstrated to date the possibility to control synthetic nanoscale devices with antibodies in a rational way^6–8^.

In nucleic acid nanotechnology synthetic nucleic acids are used as self-assembling bricks to build nanoscale devices or structural motifs of increasing complexity^9–11^. Because of their highly predictable base-pairings, low cost, ease of synthesis and biocompatibility, synthetic nucleic acids can be in fact conveniently used to rationally design self-assembling structures that can reach micrometer size but still display nanoscale quasi-Angstrom precision. Since the first examples of static DNA-based nanostructures self-assembled on a long DNA strand backbone through short DNA staples (DNA origami)^12^, a wide range of more dynamic structures, where the self-assembly is induced by specific inputs^13^, has been described together with the fine control of structural reconfiguration^14–16^. Strategies that allow the dynamic self-assembly of nanostructures regulated by nucleic acid circuits able to sense a number of environmental triggers such as temperature and pH^17,18^ or where it is possible to control self-assembly using non-equilibrium circuits^19^ have been also recently reported. Over the past decade several efforts have been devoted to find strategies to control DNA-based reactions and nanostructure assembly with biomolecular inputs^20^. For example, DNA-based nanodevices and DNA-based reactions controlled by proteins and antibodies have been proposed as possible tools for diagnostic or sensing applications^6,7,21–23^. With regards to DNA structural motifs, the control of the spatial geometry of DNA-based shapes using genetically-encoded proteins^24^ and the use of proteins, peptides and lipid bilayers as building blocks or supportive scaffolds to build DNA chimera nanostructures has been also demonstrated^25–28^. However, while these examples clearly demonstrate the versatility of DNA-based nanostructures self-assembly process, the possibility to control the assembly or disassembly of such structures with specific relevant biomolecular markers such as antibodies has not yet been demonstrated. Integrating antibody-induced regulation into DNA-based self-assembly could thus provide an attractive approach toward creating nanoscale assemblies with possible clinical applications. Motivated by the above arguments, here we demonstrate the orthogonal regulation of DNA-based circuits with specific antibodies that allow isothermal dynamic control of self-assembly and disassembly of DNA structures.

## Results

### Design of orthogonal antibody-controlled DNA circuits

**Fig. 1:**
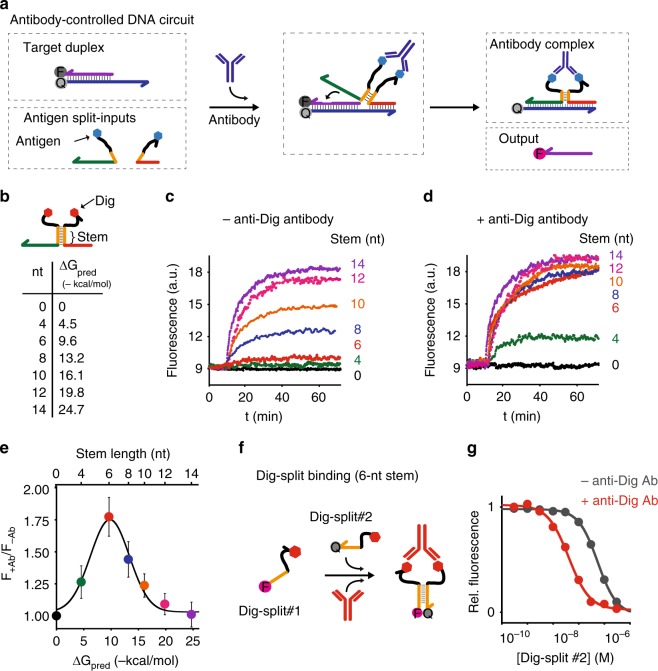
Principle and optimization of the antibody-controlled DNA circuit. **a** To engineer a strand displacement reaction controlled by antibody we have split the input strand responsible for the toehold displacement reaction into two portions (red and green) and flanked them with two complementary portions (orange) and with a 12-nt poly-T tail (black). At the two ends of such tails we have conjugated a molecule (antigen) responsible for antibody recognition. The binding of the antibody to the two antigen-conjugated split-inputs co-localizes them and induces stem formation and reconstitution of the functional input strand. **b** As a first test-bed we used Digoxigenin (Dig) as the antigen. We tested different stem lengths with various predicted ΔG values. **c** Fluorescent kinetic traces of strand displacement reactions observed by adding one of the Dig-conjugated split-inputs (60 nM) into a solution containing the other Dig-conjugated split-input (60 nM) and the optically-labeled target duplex (30 nM). **d** The same experiment described in **c** but in the presence of the specific anti-Dig antibody (300 nM). **e** Ratio between the end-point fluorescent values obtained in the presence and absence of the anti-Dig antibody vs. the predicted ΔG values of the different split-inputs employed. **f** Stem formation experiment performed by adding increasing concentrations of the Dig-conjugated split-input strand modified with a quencher (BHQ) to a solution containing the other split-input strand modified with a fluorophore (FAM) (60 nM) in the absence (gray curve) and presence (red curve) of the anti-Dig antibody (300 nM). The strand displacement experiments in this figure were performed using a target duplex labeled with a FRET couple (Cy3-Cy5) so that the displacement reaction can be easily followed through increase of the fluorescence signal. All experiments were performed in 50 mM Na_2_HPO_4_, 150 mM NaCl at pH 7.0, 25 °C. In all sketches, the 3′ ends are marked with an arrow. The experimental values represent averages of three separate measurements and the error bars reflect the standard deviations.

Our strategy to rationally regulate DNA nanostructures formation with antibodies starts from the consideration that the isothermal assembly and disassembly of such nanostructures can be induced by regulator strands that can be released by specifically designed DNA-based circuits^17,29^. To first design an antibody-controlled DNA-based circuit we took advantage of the specific Y-shaped geometry that all IgG antibodies share with two identical binding sites separated by about 6–14 nm^30–32^ and by the possibility to easily conjugate different recognition elements on the backbone of synthetic nucleic acid strands^33^. More specifically, we have started our design from a conventional toehold strand displacement reaction, which is the most employed process in DNA-based circuits^34–36^. In such reaction a DNA output strand is released from a DNA duplex target complex after the interaction with a trigger DNA (input) containing a toehold-binding domain and an invading domain. To achieve antibody control of such DNA strand displacement reaction we have taken inspiration from protein-fragment complementation assays, that use rationally designed fragments of a reporter protein to study protein/protein interactions^37,38^, and from other DNA-based systems that have used co-localization of split input strand to control strand-displacement cascades^22,23,39,40^. More specifically, we have split the input DNA strand into two separated strands (a toehold-binding strand and an invading strand) (Fig. 1a). These portions were then both flanked by two complementary stem-forming domains and by two 12-nt poly-T tails. At the end of such tails we covalently conjugated the element (i.e., the antigen) responsible for the recognition and binding to the specific target antibody. Antibody binding to the two antigen-conjugated split input strands induces their co-localization thus triggering stem formation and the reconstitution of a functional DNA input complex that can thus lead to strand displacement activation (Fig. 1a).

As a first test bed for the optimization of an antibody-powered DNA-based circuit we have conjugated the two input-forming split strands with the small-molecule hapten digoxigenin (Dig) that would be recognized by specific IgG anti-Dig antibodies. In order to observe anti-Dig-induced input strand reconstitution and to avoid non-specific leakage reaction it is important to find the optimal thermodynamic trade-off so that the DNA input-forming stem is not stable enough in the absence of the anti-Dig antibody and is instead strongly stabilized when the antibody binds to the two antigen-conjugated split strands. To achieve this we have studied DNA strand displacement reaction with the two Dig-conjugated split portions (60 nM) and a target duplex complex (30 nM) in the presence and absence of a fixed saturating concentration of the specific anti-Dig antibody (300 nM). The target duplex complex is labeled with FRET couple so that we can easily follow the strand displacement reaction by measuring in real time the fluorescence signal of the released strand (Fig. 1a). We have tested a series of Dig-conjugated split input-strands that share the same 12-nt toehold and 21-nt invading domains but differ in the length and thus thermodynamic stability of the input-forming stem. More specifically we have tested stems ranging from 4 to 14 nucleotides corresponding to predicted free energy values that go from −4.5 to −24.7 kcal/mol, respectively (Fig. 1b). To have an additional reference control we have also tested split strands that do not contain complementary stem-forming nucleotides (0-nt). We note here that the bulge created by the stem in the input strand only minimally reduces the overall efficiency of strand displacement. Under the same experimental conditions, the bulge-free input strand shows slightly better sensitivies and faster kinetics compared to the unimolecular input strand containing a 6-nt stem separating the toehold and invading portions (Supplementary Figs. 1–4). This is probably due to a less efficient invading reaction after the toehold binding due to the presence of the stem separating the toehold-binding and invading portions. As expected, because of the higher energetic contribution, in the absence of the antibody we observe significant strand displacement reaction when the stem portion is longer than 8 nucleotides and efficiencies similar to the unimolecular control input-strand when the stem is longer than 12 nucleotides (Fig. 1c). The same experiment carried out in the presence of a fixed concentration of anti-Dig antibody (300 nM) shows a completely different behavior: the co-localization induced by the binding of the antibody to the two split Dig-conjugated input-strands triggers strand displacement reaction even with a stem as short as 4 nucleotides, although with less efficiency than that of the control input (Fig. 1d). We find that a 6-nt stem leads to the strongest difference in efficiency between the absence and presence of the anti-Dig antibody (Fig. 1e). We also observe a monotonic increase of the strand displacement rate constant increasing the stem length until it plateaus with split-input strands containing stem longer than 8-nt (Supplementary Fig. 5). This behavior seems to suggest that the rate limiting step in this process is the formation of the stem rather than the binding of the antibody to the two antigen-conjugated split input strands.

**Fig. 2:**
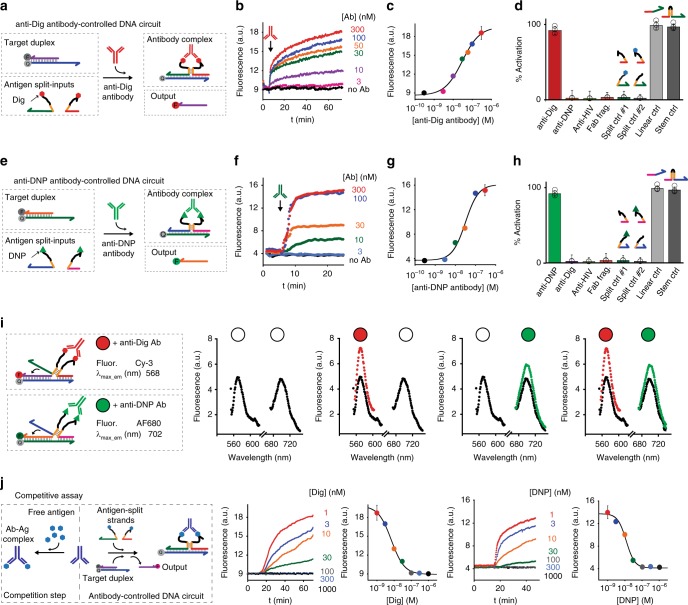
Designing orthogonal antibody-controlled DNA circuits. **a** As a first proof-of-principle of our strategy we used digoxigenin (Dig) as antigen and anti-Dig antibodies as molecular triggers of our strand displacement reaction. **b** Kinetic traces of strand displacement reaction at different concentrations of anti-Dig antibody. **c** End point values plotted vs. anti-Dig concentrations. **d** The reaction is highly specific and is only observed with the specific anti-Dig antibody. Control experiments using only a single split-input conjugated with Digoxigenin show no activation of the reaction (split ctrl. #1 and split ctrl. #2). The signal observed at saturating concentration of antibody (300 nM) is indistinguishable within error from the signal obtained with a fully linear input strand and with a unimolecular input strand containing a 6-nt stem separating the toehold-binding and invading domains. **e**–**h** Comparable efficiency and specificity can be observed using a different circuit with the two split-inputs labeled with DNP at the two ends and thus triggered by anti-DNP antibodies. **i** Orthogonal control of two antibody-controlled circuits. The two pairs of antigen-conjugated split-inputs (both at 60 nM) and the two target duplexes (both at 30 nM) are mixed in the same solution. Filled circles identify the added antibody (red = anti-Dig; green = anti-DNP). **j** Competition assays to detect free antigens (Dig, DNP). The experiments shown in this figure were performed in 50 mM Na_2_HPO_4_, 150 mM NaCl at pH 7.0, 25 °C. Strand displacement reactions were carried out in the presence of the target duplex (30 nM) and an equimolar concentration of the antigen-conjugated input-strands (60 nM). For the competition step the split-input strands were incubated in a solution containing different concentrations of the free antigen and a fixed concentration of the specific antibody (300 nM). The experimental values represent averages of three separate measurements and the error bars reflect the standard deviations. For a matter of clarity in the binding curves error bars have been depicted for only one point on each curve and represent the maximum value of standard deviation.

To further demonstrate the effect of antibody-induced co-localization we have studied the 6-nt stem formation by using the two split input-strands labeled with a fluorophore and a quencher and adding increasing concentrations of the quencher-modified split strand (split #1) to a fixed concentration (60 nM) of the fluorophore-modified split strand (split #2). In the absence of the anti-Dig antibody we observe a poor affinity (*K*_d_ = 550 ± 70 nM) between the two complementary stem portions and negligible stem formation is observed at a concentration of 100 nM of the fluorophore-conjugated split strand (Fig. 1f). As expected, the presence of the anti-Dig antibody leads to a significant improvement of the binding affinity between the two stem portions (*K*_d_ = 40 ± 10 nM) once more supporting the co-localization effect induced by antibody binding. In our next experiments we have thus employed the two Dig-conjugated split input-strands with a 6-nt stem and studied the strand displacement reaction at increasing concentration of the anti-Dig antibody.

anti-Dig antibody can efficiently induce strand-displacement reaction in a concentration-dependent fashion (Fig. 2a). The overall efficiency increases with anti-Dig antibody concentration until it saturates at about 100 nM concentration. Under the employed experimental conditions we can observe a measurable output signal from the displacement reaction at concentration of anti-Dig antibody as low as 3 nM. The strand displacement kinetic is rapid and we achieve equilibration in less than 40 min. The kinetic and sensitivity are comparable to those obtained using the unimolecular 6-nt stem control input-strand thus suggesting once again that antibody binding is rapid and does not represent the rate limiting step in this process (Supplementary Figs. 6, 7).

Because the reconstitution of the input-strand is due to the co-localization of the two antigen-conjugated split strands induced by the binding of the specific antibody, this effect is highly specific and no leakage reactions are observed in the absence of the relevant antibody (Supplementary Fig. 8). We also demonstrate that no strand displacement is observed at saturating concentrations of different non-specific antibodies and using an anti-Dig Fab fragment (that only contains a single Dig binding site) (Fig. 2d, Supplementary Fig. 9). Control experiments, employing only one of the two split strands conjugated with the antigen (while the other is unmodified) also provides a confirmation that antibody-induced co-localization is required to observe strand displacement (Fig. 2d, Split ctrl#1 and #2).

Our antibody-controlled strand displacement reaction is in principle generalizable and adaptable to other antibodies via the expedient of changing the employed recognition element. To demonstrate this we engineered a second antibody-controlled strand displacement reaction by designing a different optically-labeled duplex DNA target sequence and a new pair of split input strands conjugated with a different antigen (i.e., Dinitrophenol, DNP) (Fig. 2e). We show that only in the presence of anti-DNP antibodies the strand displacement reaction can occur. This new reaction has an efficiency, specificity, and kinetic comparable to those observed with the anti-Dig powered strand displacement reaction (Fig. 2f–h, Supplementary Figs. 10–13).

To demonstrate the possibility to use the antibody-controlled DNA-based circuits in complex samples we have repeated the same tests in 90% serum. Both the anti-Dig and anti-DNP controlled circuits work in 90% serum with an efficiency similar to that observed in pure buffer solution further demonstrating the solidity of our data and the possible sensing applications of our findings (Supplementary Figs. 14, 15).

Different antibody-controlled reactions can be regulated in the same solution in an orthogonal way without any crosstalk. To demonstrate this we have employed the same circuits studied earlier and responding to anti-Dig and anti-DNP antibodies (Fig. 2i) in the same solution. Each circuit releases an output strand labeled with a different non-interfering fluorophore (Cy-3 and AF680) so that their activation can be followed autonomously. The addition of one of the two antibodies in a solution containing both duplex targets and split input-strand pairs triggers the displacement of the relevant output DNA strand and only in the presence of both antibodies we observe the signal from both the outputs (Fig. 2i).

To further demonstrate the possible application of this approach for sensing purposes we have performed a competitive assay that allows to measure the concentration of free antigen in solution. To do this we have coupled a competition step to our antibody-induced DNA circuit (see cartoon Fig. 2j). The free antigen will compete with the DNA antigen-conjugated split-input strands for antibody binding so that the signal generated will be inversely proportional to the concentration of free antigen in solution. The results we have obtained with both anti-Dig and anti-DNP-controlled circuits demonstrate the possibility to detect Dig and DNP at low nanomolar concentration both in buffer (Fig. 2j) and 90% serum (Supplementary Figs. 16, 17).

### DNA nanostructure assembly and disassembly

**Fig. 3:**
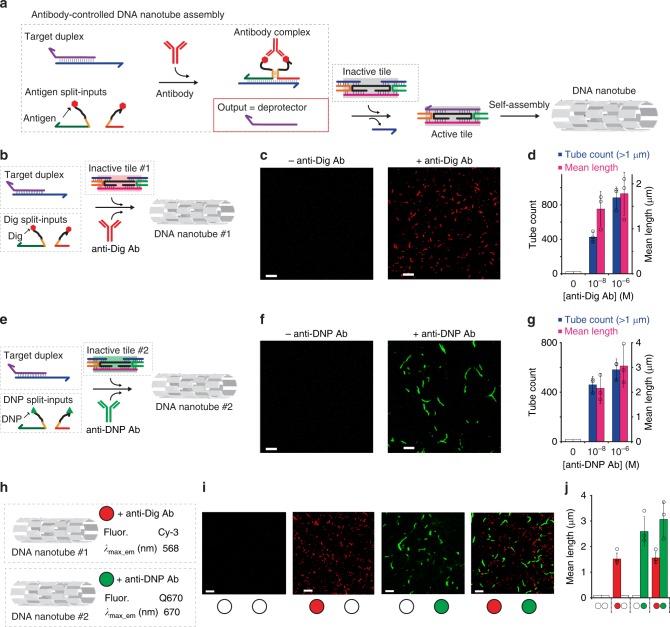
Antibody-controlled DNA nanostructure assembly. **a** Antibody-controlled DNA circuit re-engineered to trigger the assembly of DNA nanotubes. To do this we have initially employed inactive DNA double-crossover tiles^18,19,29,44^ formed by six unique DNA strands that can be activated by the antibody-controlled strand displacement output (deprotector). Activated tiles can then self-assemble into nanotubes via interactions of their sticky ends. Because one of the tile-forming strands is labeled with a fluorophore (Cy3), the formed nanotubes can be observed by fluorescence microscopy. **b** DNA circuit controlled by anti-Dig antibody leading to the formation of DNA nanotube#1. **c** Fluorescence microscopy images of nanotubes in the absence (left) and presence (right) of anti-Dig antibody (300 nM). **d** Histograms of nanotube length (mean length) and number (tube count, for nanotubes longer than 1 µm) measured from fluorescence microscopy images in the presence of different anti-Dig antibody concentrations. **e**–**g** DNA circuit controlled by anti-DNP antibody leading to the formation of DNA nanotube#2 labeled with a different fluorophore shows similar anti-DNP antibody concentration-dependent behavior. **h**, **i** Orthogonal control of the two nanotubes self-assembly in the same solution. Filled circles identify the added antibody (red = anti-Dig; green = anti-DNP). **j** Histograms of nanotube length (mean length) measured from fluorescence microscopy images. The experiments shown in this figure were performed in 1 × TAE, 12.5 mM MgCl_2_ at pH 8.0, 25 °C. Nanotubes self-assembly was carried out in the presence of the DNA circuit (target duplex, 220 nM and equimolar concentration of the antigen-conjugated input-strands, 440 nM) and a fixed concentration of the inactive tile (200 nM). No nanotubes are observed in the absence of the relevant antibody. In the histogram figures (panels **d**, **g**, **j**) the bars corresponding to mean length = 0 where no nanotubes are observed are shown as white bars for a matter of clarity. The experimental values represent averages of three separate measurements and the error bars reflect the standard deviations. Scale bars for all microscope images, 5 µm.

Antibody-controlled DNA-based circuits allow to orthogonally regulate the assembly and disassembly of responsive nucleic acid nanoscale structures using different specific antibodies as inputs (Fig. 3a). To demonstrate this we employed double-crossover DNA tiles known as DAE-E^29,41–43^ which are assembled through the interaction of six different strands. More specifically, we used a design described by Winfree and co-workers in which tiles are initially assembled into an inactive conformation that prevents self-assembly^44^. The addition of a deprotector strand to the inactive tiles causes, through a toehold strand displacement reaction, the release of the two strands responsible for inactivation. The 5-nt sticky ends of the tile that have been made accessible by such exchange reaction allow isothermal self-assembly of the tiles yielding hollow tubular structures with a maximum observed length that is on the order of few micrometers^44^. To demonstrate antibody-controlled assembly of such DNA structures we have designed a DNA-based circuit controlled by a specific anti-Dig Antibody that releases as final output a deprotector strand. Addition of the anti-Dig antibody in the presence of the inactive tiles thus causes their activation and self-assembly into nanotubes structures (Fig. 3b). To quantify nanotube length via fluorescence confocal microscopy, tiles were labeled with fluorescent dyes (Cy3) through the use of a fluorescent-labeled tile-forming DNA strand. Fluorescence confocal microscopy images of nanotubes in the absence of the specific antibody show no visible structure formation thus demonstrating once again that the antibody-controlled circuit is leakage-free. Only in the presence of anti-Dig antibody DNA nanotubes can be observed (Fig. 3c). Fluorescence confocal microscopy images were processed (see Methods section) to obtain information regarding nanotube length and number achieved at different concentrations of anti-Dig antibody (Supplementary Figs. 18, 19). A concentration-dependent behavior of nanotubes length and number could be observed (Fig. 3d). Once again, the strategy can be easily generalized to other antibodies. To demonstrate this we designed a new set of DNA tile-forming strands activated by a different deprotector strand. We have then engineered a new DNA-based circuit that releases this second deprotector only in the presence of anti-DNP antibodies (Fig. 3e, f). Also in this case, to follow the isothermal assembly of the nanotube structures we have labeled one of the tile strands with a fluorophore (Q670). A concentration-dependent nanotube formation induced by anti-DNP antibodies is observed (Fig. 3g, Supplementary Figs. 20, 21). As expected, no assembly of nanotubes can be measured in the absence of the specific antibody or in the presence of a saturating concentration of a non-specific antibody (Fig. 3g, Supplementary Fig. 22).

The already demonstrated orthogonality of the antibody-controlled DNA-based circuits offers the possibility to regulate in an orthogonal way nanostructure self-assembly. To demonstrate this we have mixed in the same solution the two different protected tiles described above and have added the two target antibodies at different combination (Fig. 3h). The results demonstrate that antibody-induced self-assembly is not affected by the presence of the other tiles and only in the presence of both antibodies we observe formation of the two different nanotubes (Fig. 3i, j, Supplementary Figs. 23–26).

**Fig. 4:**
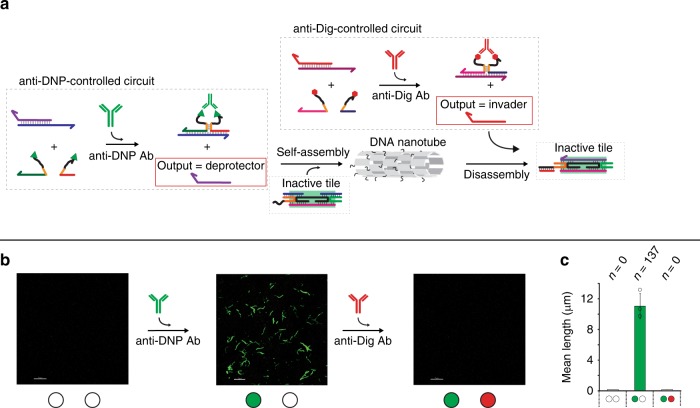
Assembly/disassembly of antibody-controlled DNA nanostructures. We have employed here tiles modified to include a single-stranded overhang, or toehold (black domain on the 5′ end of the yellow strand)^18,19^ which will be exposed on the external surface of the nanotube. An invader strand can thus bind to the toehold and displace one of the inter-tile bonds, causing the nanotubes to disassemble. **a** We have re-engineered two orthogonal antibody-controlled DNA circuits that respond to the presence of two different antibodies (anti-DNP and anti-Dig) and release two different outputs (a deprotector strand, to trigger self-assembly and an invader strand, to trigger disassembly). **b** Fluorescence confocal microscopy images of nanotubes in the absence of both antibodies (left), after addition of anti-DNP antibody (300 nM) (center) and after the addition of anti-Dig antibody (300 nM) (right). **c** Histograms of nanotube length (mean length) measured from fluorescence microscopy images. In this panel the bars corresponding to mean length = 0 where no nanotubes are observed are shown as white bars for a matter of clarity. The experiments shown in this figure were performed in 1 × TAE, 12.5 mM MgCl_2_ at pH 8.0, 25 °C. Nanotubes self-assembly was carried out in the presence of both the anti-DNP and anti-Dig DNA circuits (target duplex, 220 nM and equimolar concentration of the antigen-conjugated input-strands, 440 nM) and at a fixed concentration of the inactive tile (200 nM). The experimental values represent averages of three separate measurements and the error bars reflect the standard deviations. Scale bars for all microscope images, 5 µm.

Reversible assembly and disassembly of DNA-based nanostructures can be also achieved using different antibody inputs. To do so we have employed a modified version of the DAE-E tiles recently described by Franco and co-workers^18,19^, in which one of the tile’s sticky ends displays a 7-nt toehold domain (black domain on the 5′ end of the orange strand in Fig. 4a) that protrudes on the external surface of the assembled nanotubes. The toehold serves as a binding domain to trigger a strand invasion of the sticky ends from an invader strand causing dissociation of one of the inter-tile bonds. This eventually weakens tile-tile interactions enough to cause the nanostructure to disassemble. We have thus designed two antibody-controlled DNA-based circuits able to give as outputs a deprotector and an invader strand in the presence of anti-DNP and anti-Dig antibodies, respectively. Also in this case, to quantify nanotube length via fluorescence confocal microscopy, tiles were labeled with fluorescent dyes, then annealed at 1 µM concentration using standard protocols (see Methods section) and incubated at room temperature. The addition of anti-DNP antibodies in a solution containing both circuits and the inactive tiles causes the release of the deprotector strand and the consequent self-assembly of nanotubes (Fig. 4b, Supplementary Figs. 27, 28). The addition of anti-Dig antibodies in the same solution causes invader release and nanotubes to disassemble within minutes (Fig. 4b).

## Discussion

Here we have reported a strategy to control self-assembly and disassembly of DNA nanostructures using specific IgG antibodies as molecular inputs. The approach we propose here is versatile and in principle, generalizable to any antibody for which an antigen can be attached to a DNA anchoring strand. In support of this claim, we have demonstrated here that our approach can be extended to different triggering antibodies and the effect can be specific: two different DNA circuits can be controlled in an orthogonal way in the same solution by using two different IgG antibodies as inputs. Because it is based on the antibody-induced reconstitution of the input strand rather than on the co-localization of input and target duplex^7,45,46^, our approach for antibody-controlled DNA-based circuits appears particularly advantageous compared to other previously reported antibody-controlled DNA-based reactions^6,7^ in terms of rapid kinetic of strand displacement and for the absence of any significant leakage reaction.

Engineering DNA-circuits and self-assembly of DNA structures to be controlled by relevant biomarkers such as antibodies represents a further step towards the possible exploitation of DNA nanotechnology in the clinical field. The antibody-regulated DNA reactions and structures we have developed here may in fact open the doors to new exciting possibilities and could find application in point-of-care diagnostics, controlled drug-release and in-vivo imaging. DNA-based reactions such as toehold strand-displacement^35^ and Hybridization Chain Reaction (HCR)^39^ have been extensively used for sensing applications and providing an approach that could extend this type of systems to the detection of antibodies and antigens (through a competitive assay) in a leakage-free way could be transformatory. DNA nanostructures have also been already demonstrated to be promising tools for both diagnostic and drug-delivery applications^9,47^: controlling their assembly and disassembly with specific antibodies could be conveniently employed for such uses. While we have demonstrated the synthesis of a simple DNA nanostructure our approach could be easily extended to more complex DNA tiling approaches^48^ and DNA origami tiles^49^. Furthermore, DNA 3d assemblies could be also engineered to be responsive to different antibodies so that not only the assembly but also the spatial reconfiguration of DNA structures^16^ could be induced by these important biomolecular targets. On a more general view, the possibility to have leakage-free antibody controlled DNA circuits might represent an important advancement towards more complex synthetic genetic circuits for cell-free synthetic biology applications ^50–54^.

## Methods

### Chemicals

Reagent-grade chemicals (NaCl, MgCl_2_, Na_2_HPO_4_, Trizma hydrochloride, Acetic acid, EDTA) were purchased from Sigma-Aldrich (St Louis, Missouri) and used without further purifications. Sheep polyclonal anti-Dig antibodies were purchased from Roche Diagnostic Corporation, Germany, (cat#: 11333089001), mouse monoclonal anti-DNP antibodies were purchased from Sigma-Aldrich, USA, (cat#: D9656), murine monoclonal anti-HIV antibody was purchased from Zeptometrix Corporation, USA (cat#: 0801077), anti-Dig Fab purchased from Roche Diagnostic Corporation, (Germany) (cat#: 11214667001) and anti-DNP Fab fragments purchased from Creative Biolabs, USA, (cat#: MOB-286-F(E)). All the antibodies were aliquoted and stored at 4 °C for immediate use or at −20 °C for long-term storage.

### Oligonucleotides and DNA circuits

HPLC purified oligonucleotides were purchased from IBA, (Gottingen, Germany) or Biosearch Technologies (Risskov, Denmark). The strands forming the target duplex or the antibody-controlled DNA circuits were modified, respectively, with Cy-3 and Cy-5 or Alexa680 and BHQ-2 (black hole quencher 2). The sequences and modification schemes are reported in the Supplementary Methods.

### Fluorescent experiments

Fluorescent experiments were conducted at pH 7.0 in 50 mM Na_2_HPO_4_ buffer, 150 mM NaCl, at 25 °C in a 100 µL cuvette (total volume of the solution 100 µL). Equilibrium fluorescence measurements were obtained using a Cary Eclipse Fluorimeter, respectively, with excitation at 490 (±5) nm and acquisition at 517 (±5) nm (for DNA output strands labeled with Cy-3) or with excitation at 680 (±5) nm and acquisition at 705 (±5) nm (for DNA output strands labeled with Alexa680). Strand displacement experiments were performed using 30 nM of target duplex and 60 nM of each antigen-split input and by adding increasing concentrations of the target antibody and recording the fluorescence signal in real-time until it reached equilibrium. For the binding and activation curves performed at different concentrations of antibodies or split-input strands the observed fluorescence in the presence of different concentrations of antibody or split-input strand, *F*_[target]_, was fitted using the following four parameter logistic equation^55^:

1 $$F_{\left[ {{\mathrm{target}}} \right]} = F_{{\mathrm{min}}} + \left( {F_{{\mathrm{max}}}-F_{{\mathrm{min}}}} \right)\left[ {\left[ {{\mathrm{Target}}} \right]^{{\mathrm{nH}}}/\left( {\left[ {{\mathrm{Target}}} \right]^{{\mathrm{nH}}} + K_{1/2}^{{\mathrm{nH}}}} \right)} \right]$$

where, *F*_min_ and *F*_max_ are the minimum and maximum fluorescence values, *K*_1/2_ is the equilibrium antibody concentration at half-maximum signal, *n*_H_ is the Hill coefficient, and [Target] is the concentration of the specific antibody or split-input strand added. This model is not necessarily physically relevant, but it does a good (empirical) job of fitting effectively bi-linear binding curves such as those we obtain for our systems.

### Nanotubes assembly and disassembly

The protected inactive tiles for all the systems were prepared as reported elsewhere^44^. Briefly, each tile strand was mixed at 1 µM (final concentration) in Tris Acetate-EDTA (TAE)/Mg^2+^ (TAE buffer 1×, 12.4 mM MgCl_2_, pH 8) buffer and annealed with a Bio-Rad Mastercycler Gradient thermocycler by heating the solution (50 µL) to 90 °C, and cooling it to 20 °C at a constant rate over a 24 h period. For antibody-induced self-assembly of the nanotubes the so prepared protected tiles were mixed with the anti-Dig or anti-DNP DNA circuit releasing the deprotector strand (see Supplementary Methods). The concentration of the protected tile was 200 nM while the concentration of the duplex DNA target and the antigen-conjugated split input strands was 220 nM and 440 nM, respectively. After a period of incubation (20 min) the relevant antibody was added and the solution was let to react at constant temperature for 24 h unless otherwise noted (25 °C). For antibody-induced self-assembly and disassembly of the nanotubes (Fig. 4) the protected tiles were mixed with both the relevant anti-Dig and anti-DNP DNA circuits releasing the deprotector and invader strands (see Supplementary Methods). The concentration of the protected tile was 200 nM while the concentration of both the duplex DNA targets was 220 nM and that of both pairs of antigen-conjugated split input strands was 440 nM. After a period of incubation (20 min) anti-DNP antibody was added (300 nM) and the solution was let to react at constant temperature for 24 h (25 °C). After imaging the formed nanotubes, anti-Dig antibodies (300 nM) were added and the solution was let to react at constant temperature for 24 h (25 °C). All control experiments in the absence of the specific antibody or with a non-specific antibody where performed letting the solution to react for 24 h (25 °C).

### Fluorescence imaging of nanotubes

For fluorescence microscopy imaging the central strand of each tile (t4, see sequence in Supplementary Methods) was labeled at the 3′ or 5′ end with a fluorophore (Q570, Q670, Cy3). A confocal laser scanning microscope Olympus FV-1000 was used. The emitted photons were collected by a ×60, oil objective. A 2 μL drop of the mixture reaction (50 nM) was deposited between a clean microscope slide and a coverslip. Nanotube length distributions and counts were quantified by image metrology using the SPIP software (www.imagemet.com).

### Reporting summary

Further information on research design is available in the Nature Research Reporting Summary linked to this article.

## Supplementary information

Supplementary Information

Reporting Summary

## Data Availability

The source data underlying the Figures in the main text are provided as a Source Data file. Other data that support the findings of this study and underlying the Supplementary Figures are available from the corresponding author upon reasonable request.

## Source data

Source data
