# Publisher Correction: Orthogonal regulation of DNA nanostructure self-assembly and disassembly using antibodies

**DOI:** 10.1038/s41467-019-13971-z

**Published:** 2020-01-13

**Authors:** Simona Ranallo, Daniela Sorrentino, Francesco Ricci

**Affiliations:** Chemistry Department, University of Rome, Tor Vergata, Via della Ricerca Scientifica, 00133 Rome, Italy

**Keywords:** Supramolecular chemistry, DNA nanotechnology, Nanobiotechnology

Correction to: *Nature Communications* 10.1038/s41467-019-13104-6, published online 03 December 2019.

The original version of this Article contained an error in the legend of Fig. 4b. The text for Fig. 4b which should be “Fluorescence confocal microscopy images of nanotubes in the absence of both antibodies (left), after addition of anti-DNP antibody (300 nM) (center) and after the addition of anti-Dig antibody (300 nM) (right)” was incorrectly given as “b Fluorescence confocal microscopy images of nanotubes in the absence of both antibodies (left), after addition of anti-Dig antibody (300 nM) (center) and after the addition of anti-DNP antibody (300 nM) (right).” This has been corrected in both the PDF and HTML versions of this Article.

