# Supplementary material for: Orthogonal regulation of DNA nanostructure self-assembly and disassembly using antibodies
Source: Nat Commun. 2019 Dec 3;10:5509. doi: 10.1038/s41467-019-13104-6 (PMC6890650; doi:10.1038/s41467-019-13104-6)
Supplement: Supplementary file 1 — Supplementary Information [file 41467_2019_13104_MOESM1_ESM.pdf]

Supplementary Information:

Orthogonal regulation of DNA nanostructure self-assembly and disassembly using antibodies

Simona Ranallo, Daniela Sorrentino and Francesco Ricci

Supplementary Methods

DNA Sequences

HPLC purified oligonucleotides were purchased from IBA, (Gottingen, Germany) or Biosearch Technologies (Risskov, Denmark).

Antibody-controlled circuit sequences

All sequences for the DNA circuits were designed using Nupack.[1]

Anti-Dig controlled DNA strand displacement circuit:

Name	Sequence
S1	5'-(Cy5) CCT CAT CAT CAT ATA CGT CAC CTA TCC CAT TCT
S2	5'-GTG ACG TAT ATG ATG ATG AGG -(Cy3)
Linear control	5'-**AGA ATG GGA TAG** - *GTG ACG TAT ATG ATG ATG AGG*
bulge 6nt-stem control	5'-**AGA ATG GGA TAG** TT GTCTGC TTT TTT TTT T GCAGAC TT *GTG ACG TAT ATG AT G ATG AGG*
Dig_Inv21_Stem14	5'-(Dig) TT TTT TTT TTT GAC AGC AGA CGA TG TT *GTG ACG TAT ATG ATG AGG*
Dig_TH12_Stem14	5'-**AGA ATG GGA TAG** TT CA TCG TCT GCT GTC TTT TTT TTT TTT -(Dig)
Dig_Inv21_Stem12	5'-(Dig) TT TTT TTT TTT CAG CAG ACG ATG TT *GTG ACG TAT ATG ATG ATG AGG*
Dig_TH12_Stem12	5'-**AGA ATG GGA TAG TT** CAT CGT CTG CTG TTT TTT TTT TT -(Dig)
Dig_Inv21_Stem10	5'-(Dig) TT TTT TTT GAC AGC AGA C TT *GTG ACG TAT ATG ATG ATG AGG*
Dig_TH12_Stem10	5'-**AGA ATG GGA TAG** TT G TCT GCT GTC TTT TTT TT (Dig)
Dig_Inv21_Stem8	5'-(Dig) TT TTT TTT C AGC AGA C TT *GTG ACG TAT ATG ATG ATG AGG*
Dig_TH12_Stem8	5'-**AGA ATG GGA TAG** TT G TCT GCT GTTT TTT TT- (Dig)
Dig_Inv21_Stem6	5'-(Dig) TTT TTT TTT TTT GCA GAC TT *GTG ACG TAT ATG ATG ATG AGG*
Dig_TH12_Stem6	5'-**AGA ATG GGA TAG** TT GTC TGC TTT TTT TTT TTT (Dig)

Dig_Inv21_Stem4	5'-(Dig) TTT TTT TTT TTT A GAC TT *GTG ACG TAT ATG ATG ATG AGG*
Dig_TH12_Stem4	5'-**AGA ATG GGA TAG** TT GTC T TTT TTT TTT TTT -(Dig)
Dig_Inv21_Stem0	5'-(Dig)TTT TTT TTT TTT TGG GTG TT *GTG ACG TAT ATG ATG ATG A*

In the above sequences the portion in **bold** represents the toehold domain, while the portion in *italics* represents the invading domain. The underlined bases represent the stem-forming portions. In the antigen-conjugated split input strands Dig was introduced via EDC/NHS coupling to an amine attached via a 5-carbon linker on the 5' end or on the 3' end.

Anti-DNP controlled DNA strand displacement circuit:

**Name**	**Sequence**
S1	5'-(AF680) CCA ATA TCA TCA AGA GCG TAC GAT TCG TGA GTA
S2	5'-GTA CGC TCT TGA TGA TAT TGG-(BHQ2)
Linear control	5'-**TAC TCA CGA ATC** *GTA CGC TCT TGA TGA TAT TGG*
Unimolecular 6nt-stem control	5'-**TAC TCA CGA ATC** TT GTC TGC TTT TTT TTT T GCA GAC TT *GTA CGC TCT TGA TGA TAT TGG*
DNP_Inv21_Stem6	5'-(DNP)TTT TTT TTT TTT GCA GAC TT *GTA CGC TCT TGA TGA TAT TGG*
DNP_TH12_Stem6	5'- **TAC TCA CGA ATC** TT GTC TGC TTT TTT TTT TTT-(DNP)

In the above sequences the portion in **bold** represents the toehold domain, while the portion in *italics* represents the invading domain. The underlined bases represent the stem-forming portions. In the antigen-conjugated split input strands DNP was attached via a triethylene glycol (TEG) spacer arm on either the 5' or the 3' terminus of the appropriate oligonucleotide.

Antigen-conjugated split DNA strand binding curves

To investigate the stem-formation between the two antigen-split inputs we have employed the following fluorophore and quencher modified sequences:

**Name**	**Sequence**
Dig-Split #2	5'-(Dig) TTT TTT TTT TTT GCA GAC-(BHQ1)
Dig-Split #1	5'- (FAM) GTC TGC TTT TTT TTT TTT-(Dig)

Antibody-controlled nanotubes sequences

To design antibody-controlled nanotubes, we employed the DAE-E tile type[2-4] which consists of five distinct strands of DNA; the acronym indicates Double crossover, Antiparallel helices, Even number of half-turns between intra-tile crossovers, and Even number of helical half-turns between inter-tile crossovers. The formation of individual tiles requires thermal annealing (see Methods section); however, nanotube self-assembly proceeds at room temperature[2,4-6]. Sequences for the tiles were taken from previous papers[2,4] while sequences for the antibody-controlled circuits were designed from scratch using Nupack.[1]

Anti-Dig Antibody-controlled DNA Nanotubes:

**Name**	**Sequence**
t1_1	5'-ATA CCA TAG ATC CTG ATA GC
t2_1	5'-AGC AAC CTG AAA CCA GAA TT
t3_1	5'-GAA TTC TAC TCG TGG ATC TAT GGT AT
t4_1 (Q570)	5'-AGA ATT GCG TCG TGG TTG CTA GGT CTC GCT ATC ACC GAT GTG -Q570
t5_1	5'-AAT TCT GGT TTC ACC TTA ACG ATA CC
t6_1	5'-CGT TAA GGA CGA CGC AAT TCT CAC ATC GGA CGA GTA G
Dig_Substrate	5'-CAT GGT GAG GGA AAG AAA AGA GGG TGG TTT CAG GTT

	GCT AG G TCT C
Dig_Deprotector	5’-ATA GAT CCT GAT AGC GAG AC C TAG CAA CCT GAA ACC A
Dig_TH	5’-(Dig) TTT TTT TTT TTT GGC GAT TT **CCC TCT TTT CTT TCC C**
Dig_Inv	5’- *AGA TCT AGC AAC CTG AAA CCA* TT ATC GCC TTT TTT TTT TTT-(Dig)

In the above sequences the strands *t1_1, t2_1, t3_1, t4_1, t5_1* and *t6_1* are the strands forming the protected inactive tile. Strand *t4_1* has been used conjugated to a fluorophore (Quasar570) at the 3’ end. The strands *Dig_substrate* and *Dig_deprotector* are two complementary strands forming the target duplex for the Anti-Dig strand displacement reaction. The strands *Dig_TH* and *Dig_Inv* are the two split input strands (toehold and invading) conjugated with Digoxigenin.

Anti-DNP Antibody-controlled DNA Nanotubes:

**Name**	**Sequence**
t1_2	5’- TAC CTC TCA GTG GAC AGC CG
t2_2	5’- GCG TTG GAC GAA ACT GTC TG
t3_2	5’- GTC TGG TAG AGC ACC ACT GAG AGG TA
t4_2 (Q670)	5’- TCC AGA ACG GCT GTG GCT AAA CAG TAA CCG AAG CAC CAA CGC T-Q670
t5_2	5’- CAG ACA GTT TCG TGG TCA TCG TAC CT
t6_2	5’- CGATGACCTGCTTCGGTTACTGTTTAGCCTGCTCTAC
DNP_Substrate	5’- TGG TGA TTG GTG AGT TAG TTT CGT CCA ACG CTC CAG AA
DNP_Deprotector	5’- CTC AGT GGA CAG CCG TTC TGG AGC GTT GGA CGA AAC T
DNP_TH	5’- (DNP)- TTT TTT TTT TTT TGC CCA TT **AAC TCA CCA A TCA CCA**
DNP_Inv	5’- *TCT GGA GCG TTG GAC GAA ACT* TT TGG GCA TTT TTT TTT TTT – (DNP)

In the above sequences the strands *t1_2, t2_2, t3_2, t4_2, t5_2* and *t6_2* are the strands forming the protected inactive tile. Strand *t4_2* has been used conjugated to a fluorophore (Quasar670) at the 3' end. The strands *DNP_substrate* and *DNP_deprotector* are two complementary strands forming the target duplex for the Anti-DNP strand displacement reaction. The strands *DNP_TH* and *DNP_Inv* are the two split input strands (toehold and invading) conjugated with DNP.

DNA nanotube circuits for assembly and disassembly:

We employed a version of the nanotubes designed by Franco and co-workers by modifying the sequences reported previously.[2] Briefly, the tiles of the nanotubes are redesigned to include an actuation domain (see bold nucleotides in strand t3_S3) that allows to activate or deactivate their capacity to self-assemble. These actuation domains are single stranded toeholds exposed by design on the external nanotube surface.

**Name**	
DNP_deprotector	5'-CTC AGT GGA CAG CCG TTC TGG AGC GTT GGA CGA AAC T
DNP_substrate	5'- TGG TGA TTG GTG AGT TAG TTT CGT CCA ACG CTC CAG AA
DNP_TH	5'- (DNP)- TTT TTT TTT TTT <u>TGC CCA</u> TT **AAC TCA CCA A TCA CCA**
DNP_Inv	5'- *TCT GGA GCG TTG GAC GAA ACT* TT <u>TGG GCA</u> TTT TTT TTT TTT – (DNP)
t1_S3	5'-TAC CTC TCA GTG GAC AGC CG
t2_S3	5'-GCG TTG GAC GAA ACT GTC TG
t3_S3	5'-**TGG TAT T** GTC TGG TAG AGC ACC ACT GAG AGG TA
t4_S3 (Cy3)	5'-(Cy3) T CCA GAA CGG CTG TGG CTA AAC AGT AAC CGA AGC ACC AAC GCT
t5_S3	5'-CAG ACA GTT TCG TGG TCA TCG TAC CT
t6_S3	5'-CGA TGA CCT GCT TCG GTT ACT GTT TAG CCT GCT CTA C
Dig_invader	5'-ACC AGA C**AA TAC CA**C ACA GAA

Dig_Substrate	5’-CAT GGT GAG GGA TAC ATT AGTG GGT TTC TGT GTG GTA TTG TCT GGT
DIS_Dig_TH	5’-(Dig) TTT TTT TTT TTT GGC GAT TT **CCC ACT AAT GTA TCCC**
DIS_Dig_Inv	5’-*ACC AGA CAA TAC CAC ACA GAA* TT ATC GCC TTT TTT TTTTTT-(Dig)

In the above sequences the strands *t1_3, t2_3, t3_3, t4_3, t5_3* and *t6_3* are the strands forming the protected inactive tile. Strand *t4_3* has been used conjugated to a fluorophore (Cy3) at the 5’ end. The strands *DNP_substrate* and *DNP_deprotector* are two complementary strands forming the target duplex for the Anti-DNP controlled circuit (see Figure 4). The strands *DNP_TH* and *DNP_Inv* are the two split input strands (toehold and invading) conjugated with DNP forming the input strand in the presence of Anti-DNP antibodies (see Figure 4). The strands *Dig_substrate* and *Dig_invader* are two complementary strands forming the target duplex for the Anti-Dig controlled circuit (see Figure 4). The strands *Dig_TH* and *Dig_Inv* are the two split input strands (toehold and invading) conjugated with Dig forming the input strand in the presence of Anti-Dig antibodies (see Figure 4). In these sequences the portion in **bold** represents the Toehold domain, while the portion in *italics* represents the invading domain. The underlined bases represent the stem-forming portions. Strand t3_S3 contains a 7-base portion (bold in the sequence) that allows disassembly in the presence of the invader strand (Dig_invader) released by the Anti-Dig antibody.

Supplementary Figures

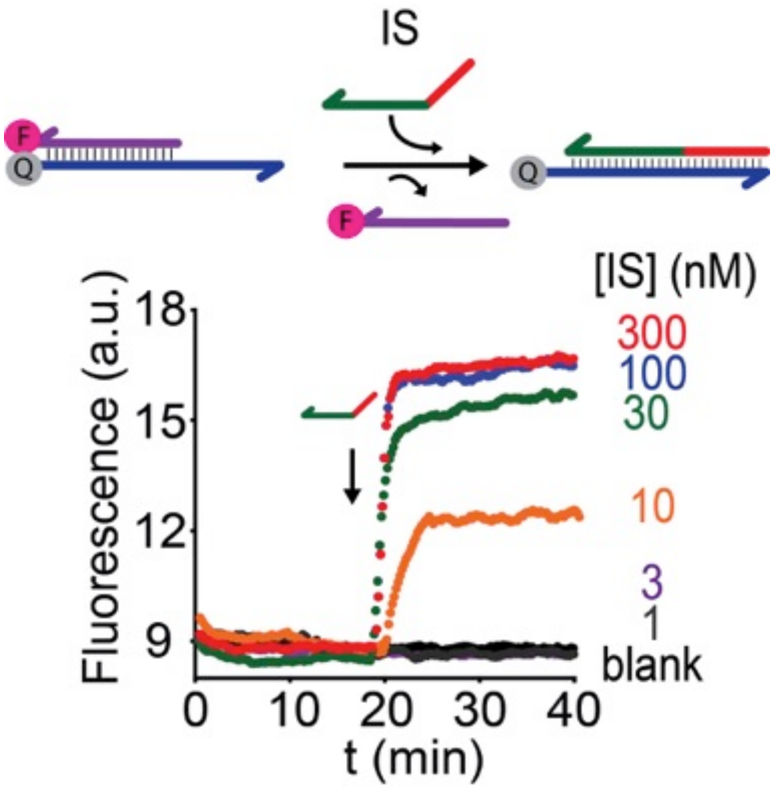

Supplementary Figure 1. Activation of toehold strand displacement reaction of Anti-Dig antibody controlled DNA circuit by using a linear control input strand. Strand displacement is followed by fluorescence measurements obtained in a solution of target duplex (30 nM) after the addition of different concentrations of the linear control input strand in a 50 mM phosphate buffer, 150 mM NaCl, pH 7.0 at 25°C.

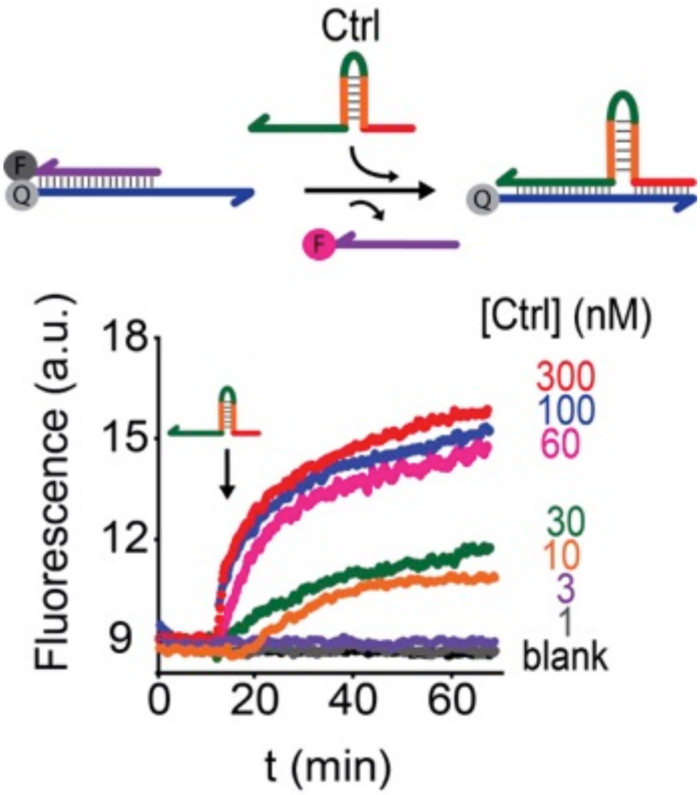

Supplementary Figure 2. Activation of toehold strand displacement reaction of Anti-Dig antibody controlled DNA circuit by using a bulge 6nt-stem control input strand. Strand displacement is followed by fluorescence measurements obtained in a solution of target duplex (30 nM) after the addition of the bulge 6nt-stem control input strand at different concentrations in a 50 mM phosphate buffer, 150 mM NaCl, pH 7.0 at 25°C.

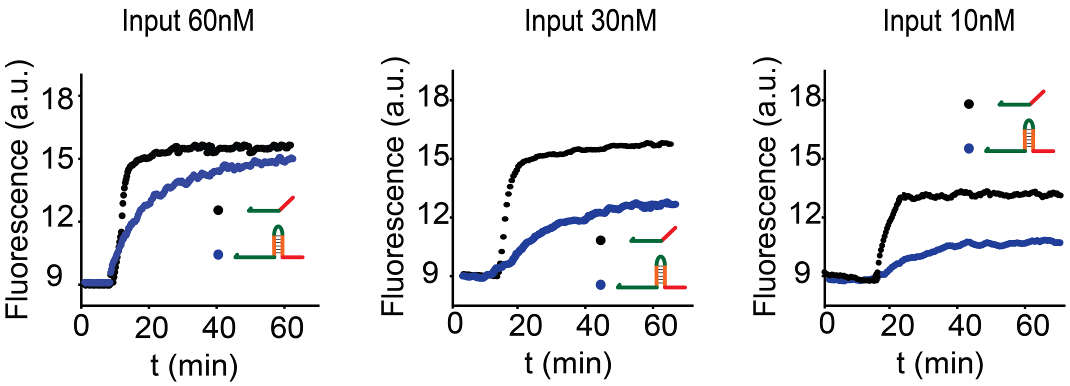

Supplementary Figure 3. Kinetic comparison of the toehold strand displacement reaction using the Anti-Dig antibody controlled DNA circuit with a linear control (black) and a bulge 6nt-stem control input strand (blue). Here strand displacement is followed by fluorescence measurements obtained in a solution of target duplex (30 nM) after the addition of the bulge 6nt-stem control input strand or the linear control input strand in a 50 mM phosphate buffer, 150 mM NaCl, pH 7.0 at 25°C.

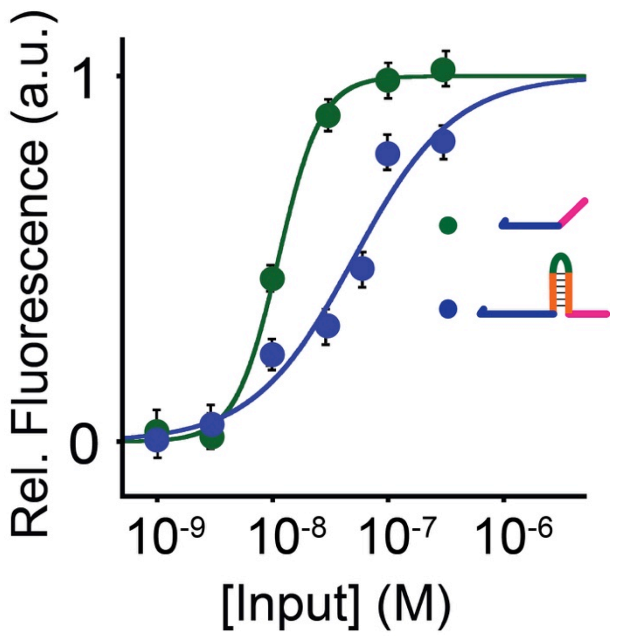

Supplementary Figure 4. Comparison of the sensitivity of the toehold strand displacement reaction using a conventional control input strand (green) and a bulge 6nt-stem control input strand (blue). Curves obtained by fitting the fluorescence signals obtained at different concentrations of the two input strands in a 50 mM phosphate buffer, 150 mM NaCl, pH 7.0 at 25°C. The experimental values represent averages of three separate measurements and the error bars reflect the standard deviations.

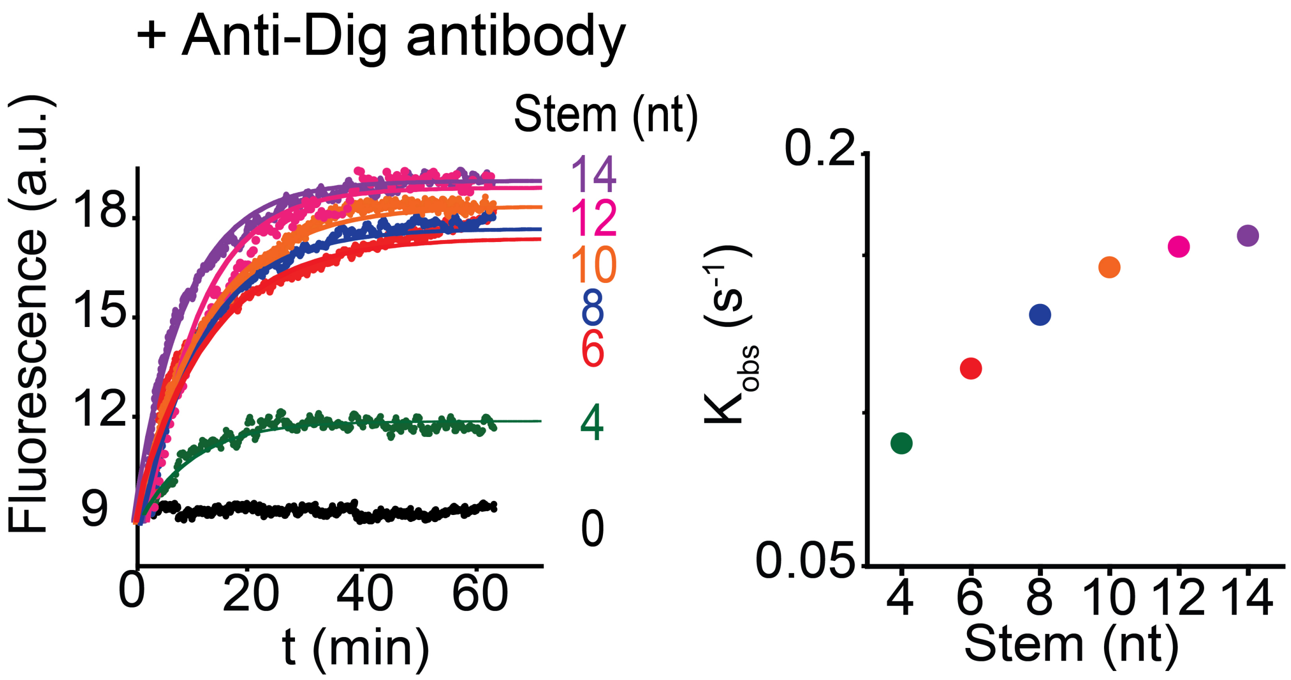

Supplementary Figure 5. (left) Fluorescent kinetic traces of strand displacement reactions observed in the presence of the two Dig-conjugated split-inputs (60 nM) and the optically-labelled target duplex (30 nM) after adding the specific Anti-Dig antibody (300 nM). (right) Apparent first-order rate constants (k_{obs}, s^{-1}) obtained by fitting the kinetic traces using a single exponential.

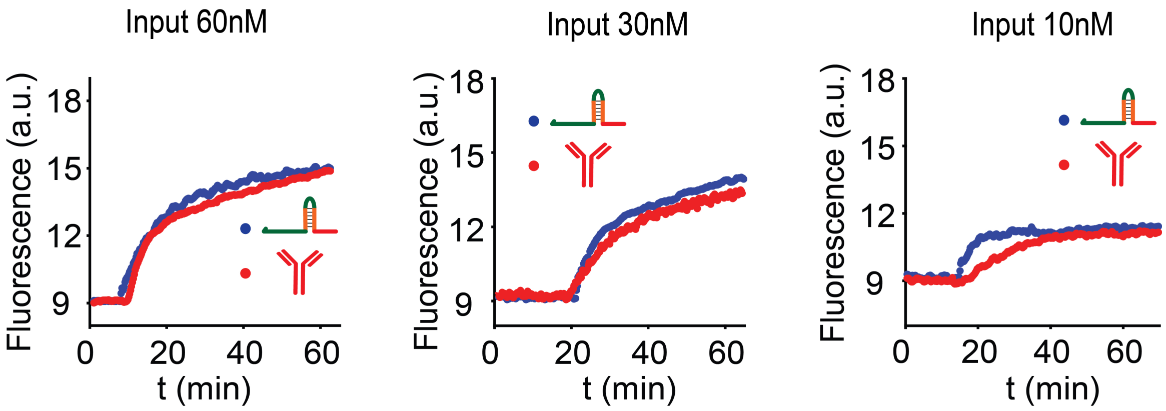

Supplementary Figure 6. Kinetic comparison of the toehold strand displacement reaction using the Anti-Dig antibody controlled DNA circuit with a bulge 6nt-stem control input strand (blue) and the Anti-Dig antibody (red). Here strand displacement is followed by fluorescence measurements obtained in a solution of target duplex (30 nM) in a 50 mM phosphate buffer, 150 mM NaCl, pH 7.0 at 25°C. The experiments in the presence of antibodies have been performed by using different concentrations of the split-input strands (indicated in each panel) and a saturating concentration of the Anti-Dig antibody (i.e. 300 nM).

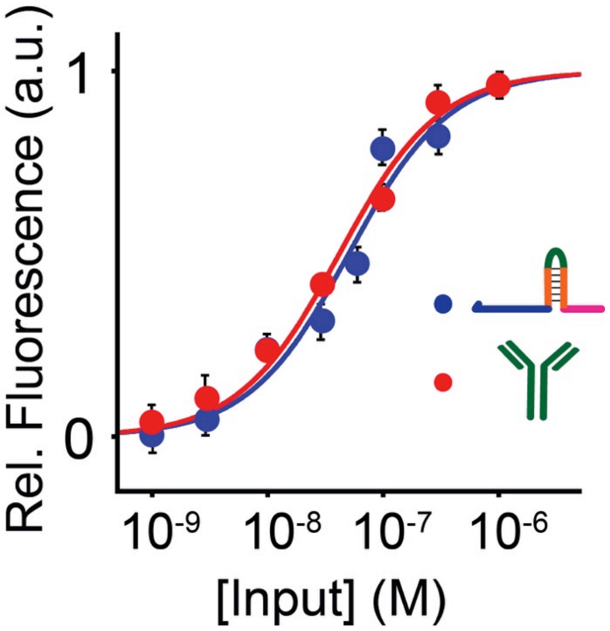

Supplementary Figure 7. Comparison of the sensitivity of the toehold strand displacement reaction using a bulge 6nt-stem control input strand (blue) and the Anti-Dig antibody (red). Curves obtained by fitting the fluorescence signals obtained at different concentrations of the input strand in a solution of target duplex (30 nM) in a 50 mM phosphate buffer, 150 mM NaCl, pH 7.0 at 25°C. The experiments in the presence of antibodies have been performed by using different concentrations of the split-input strands (indicated in the x-axis) and a saturating concentration of the Anti-Dig antibody (i.e. 300 nM). The experimental values represent averages of three separate measurements and the error bars reflect the standard deviations.

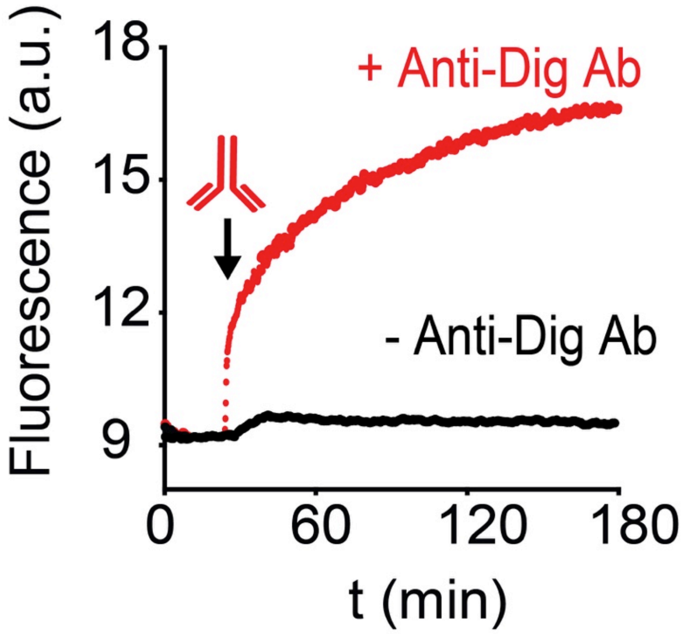

Supplementary Figure 8. Kinetic experiment using the Anti-Dig antibody controlled DNA circuit in the presence and absence of the Anti-Dig antibody (300 nM). Here strand displacement is followed by fluorescence measurements obtained in a solution of target duplex (30 nM) and split input strands (60 nM) in a 50 mM phosphate buffer, 150 mM NaCl, pH 7.0 at 25°C.

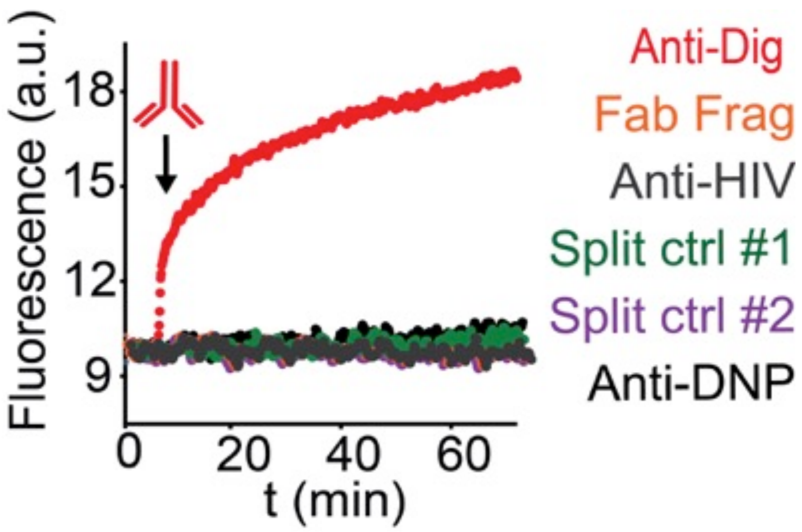

Supplementary Figure 9. Specificity experiment of the toehold strand displacement reaction using the Anti-Dig antibody controlled DNA circuit and different targets: Anti-Dig Fab fragment (Fab Frag), Anti-HIV antibody (Anti-HIV), Anti-Dig antibody added to a solution containing both split input strands but where split #1 or split #2 are not modified with Dig (split ctrl#1, split ctrl#2), Anti-DNP antibody (Anti-DNP). Here strand displacement is followed by fluorescence measurements obtained in a solution of target duplex (30 nM) and split input strands (60 nM) after the addition of the relevant antibody in a 50 mM phosphate buffer, 150 mM NaCl, pH 7.0 at 25°C.

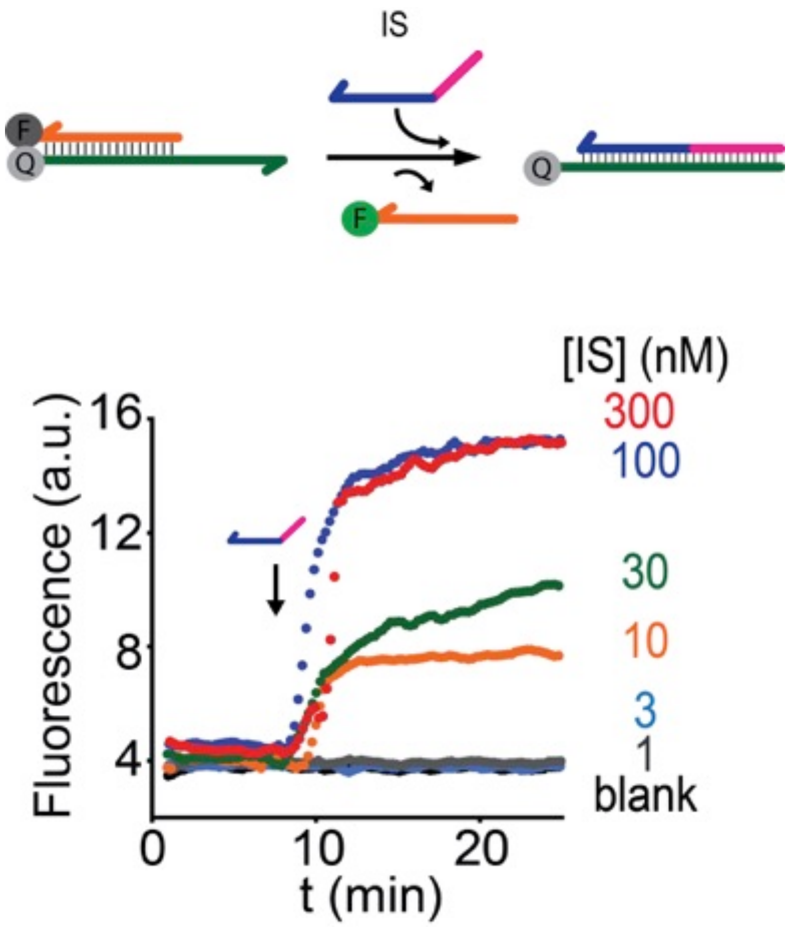

Supplementary Figure 10. Activation of toehold strand displacement reaction of Anti-DNP antibody controlled DNA circuit by using a linear control input strand. Here strand displacement is followed by fluorescence measurements obtained in a solution of target duplex (30 nM) after the addition of different concentrations of the linear control input strand in a 50 mM phosphate buffer, 150 mM NaCl, pH 7.0 at 25°C.

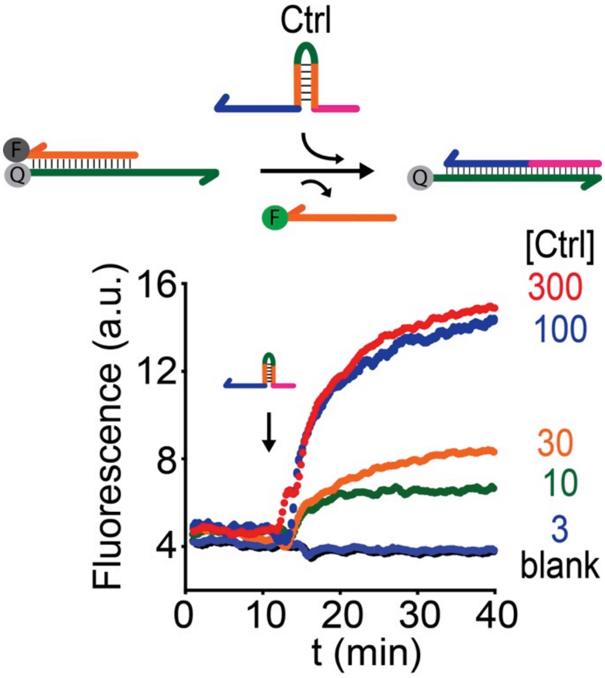

Supplementary Figure 11. Activation of toehold strand displacement reaction of Anti-DNP antibody controlled DNA circuit by using a bulge 6nt-stem control input strand. Strand displacement is followed by fluorescence measurements obtained in a solution of target duplex (30 nM) after the addition of the bulge 6nt-stem control input strand at different concentrations in a 50 mM phosphate buffer, 150 mM NaCl, pH 7.0 at 25°C.

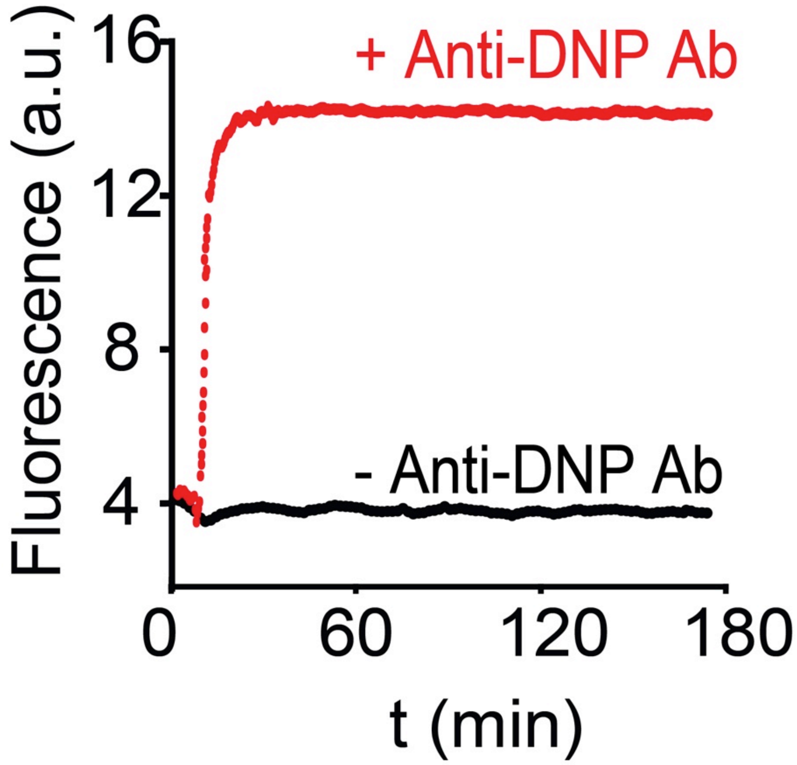

Supplementary Figure 12. Kinetic experiment using the Anti-DNP antibody controlled DNA circuit in the absence and presence of the Anti-DNP antibody (300 nM). Here strand displacement is followed by fluorescence measurements obtained in a solution of target duplex (30 nM) and split input strands (60 nM) in a 50 mM phosphate buffer, 150 mM NaCl, pH 7.0 at 25°C.

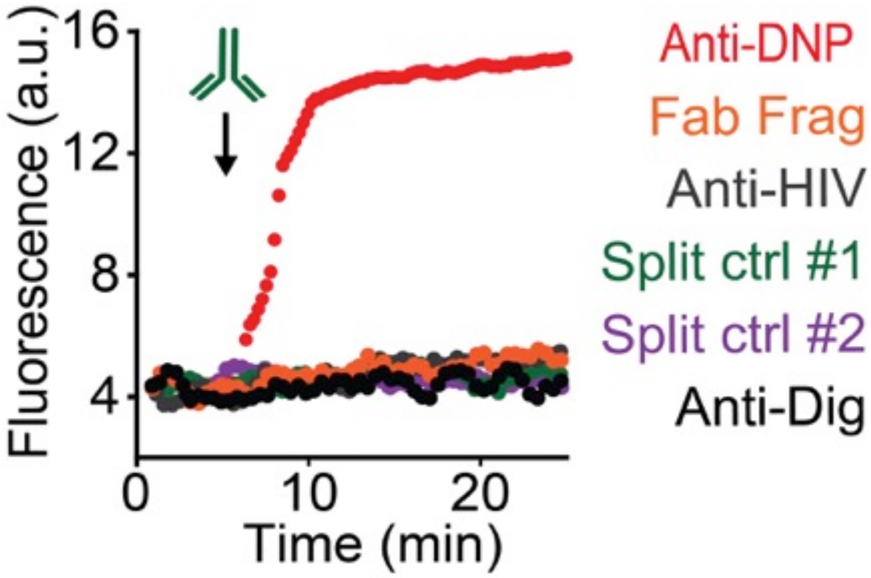

Supplementary Figure 13. Specificity experiment of the toehold strand displacement reaction using the Anti-DNP antibody controlled DNA circuit and different targets: Anti-DNP Fab fragment (Fab Frag), Anti-HIV antibody (Anti-HIV), Anti-DNP antibody added to a solution containing both split input strands but where split #1 or split #2 are not modified with Dig (split ctrl#1, split ctrl#2), Anti-Dig antibody (Anti-Dig). Here strand displacement is followed by fluorescence measurements obtained in a solution of target duplex (30 nM) and split input strands (60 nM) after the addition of the relevant antibody in a 50 mM phosphate buffer, 150 mM NaCl, pH 7.0 at 25°C.

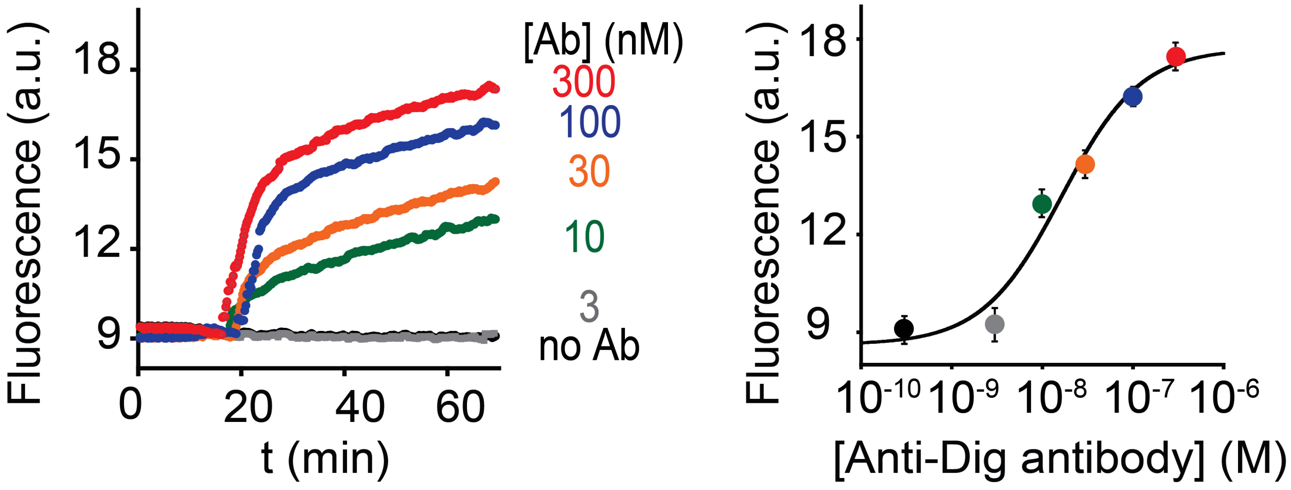

Supplementary Figure 14. Strand displacement reaction of Anti-Dig antibody controlled DNA circuit in 90% serum solution. The experimental values represent averages of three separate measurements and the error bars reflect the standard deviations.

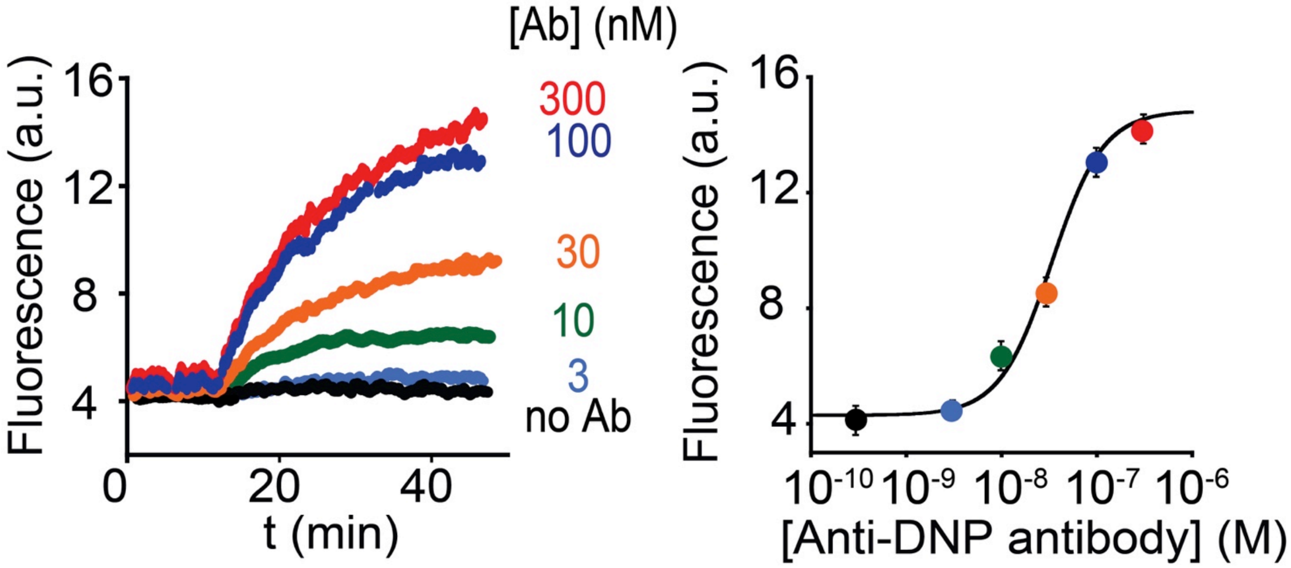

Supplementary Figure 15. Strand displacement reaction of Anti-DNP antibody controlled DNA circuit in 90% serum solution. The experimental values represent averages of three separate measurements and the error bars reflect the standard deviations.

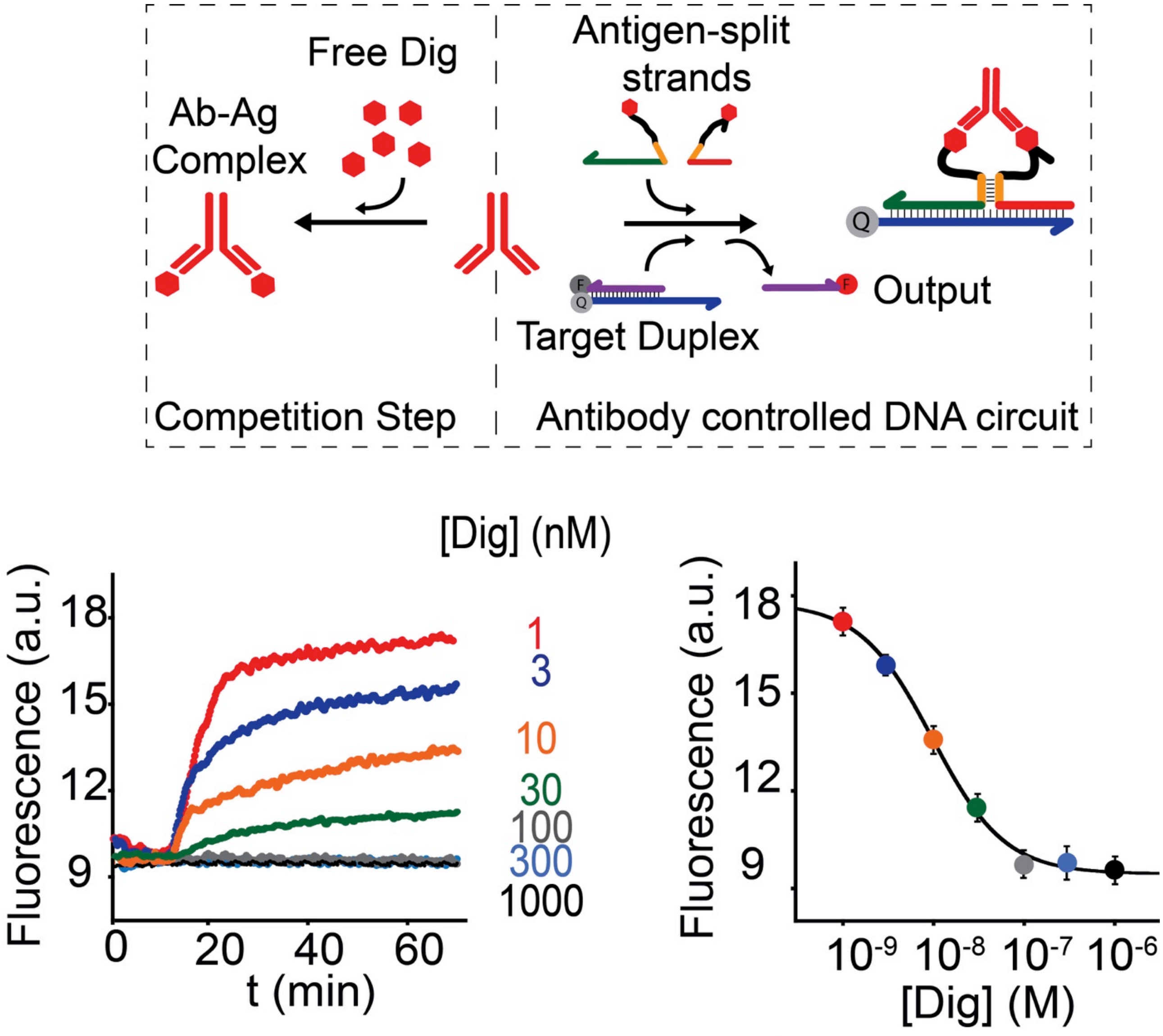

Supplementary Figure 16. Competition assay with Anti-Dig antibody controlled DNA circuit for the detection of free Dig in 90% serum solution. The experimental values represent averages of three separate measurements and the error bars reflect the standard deviations.

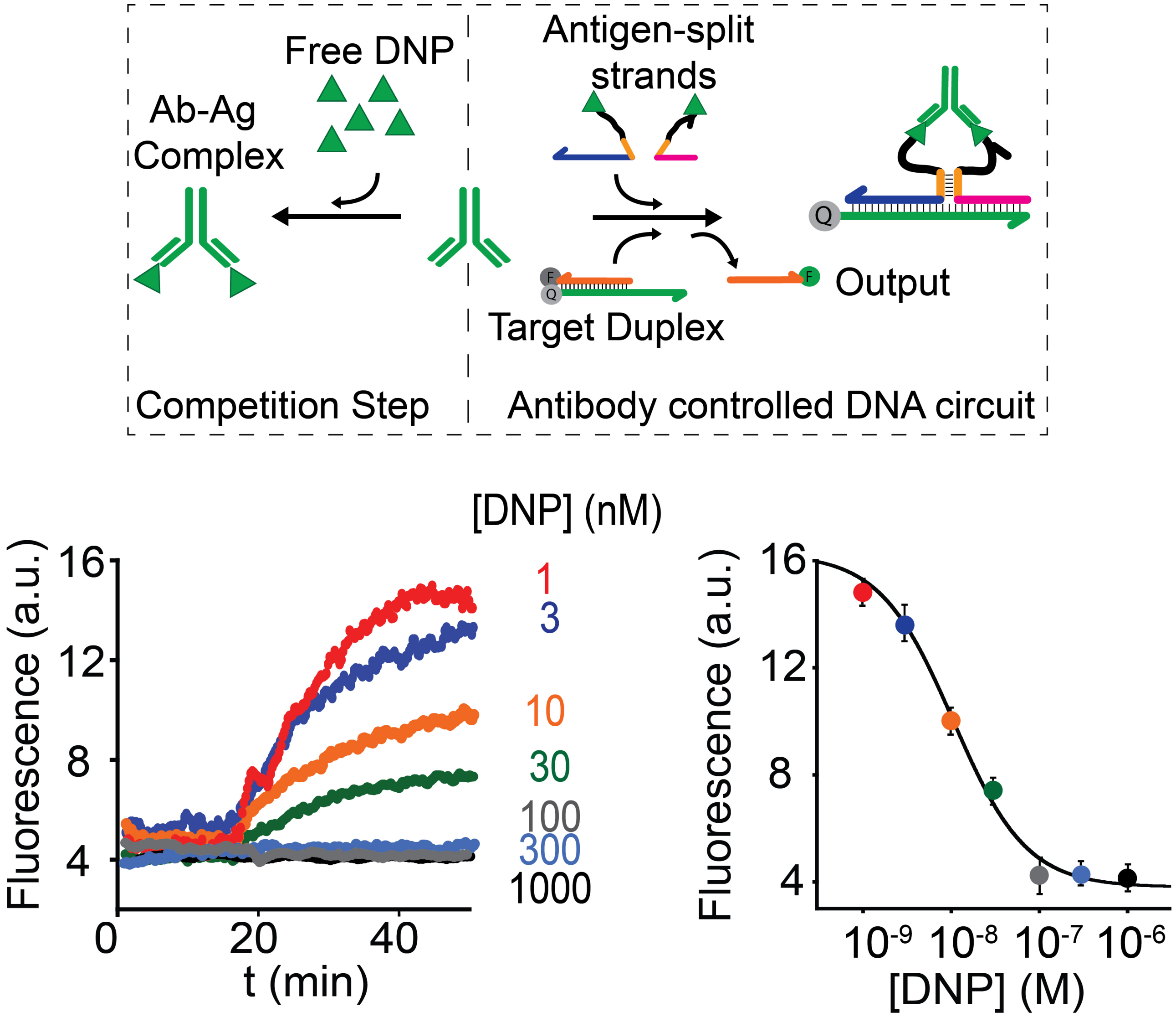

Supplementary Figure 17. Competition assay with Anti-DNP antibody controlled DNA circuit for the detection of free DNP in 90% serum solution. The experimental values represent averages of three separate measurements and the error bars reflect the standard deviations.

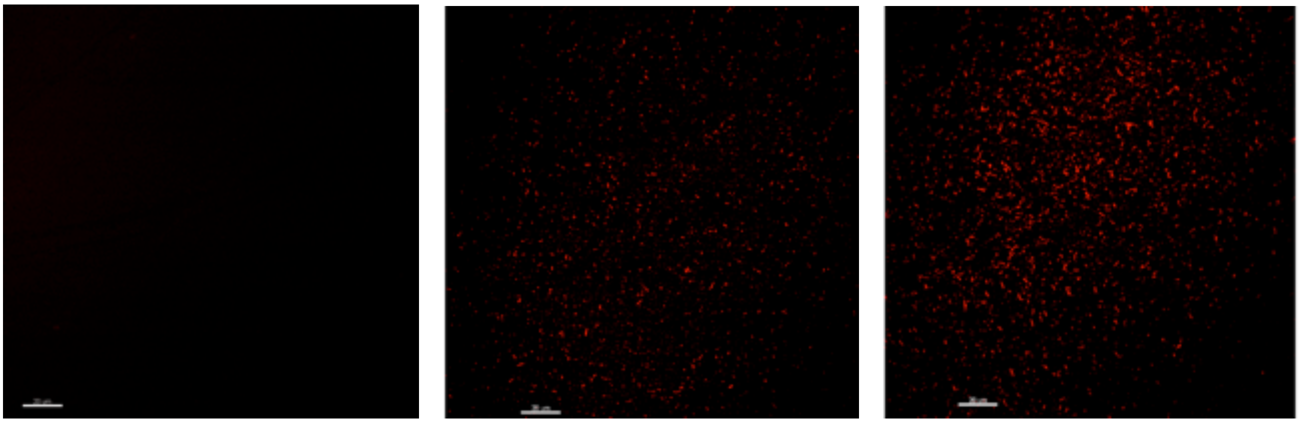

Supplementary Figure 18. Fluorescence microscopy images of nanotubes in the absence (left) and presence of Anti-Dig antibody (10 nM, center; 1 μM, right). See legend of Figure 3 for experimental details. Scale bars for these microscope images, 20 μm.

*

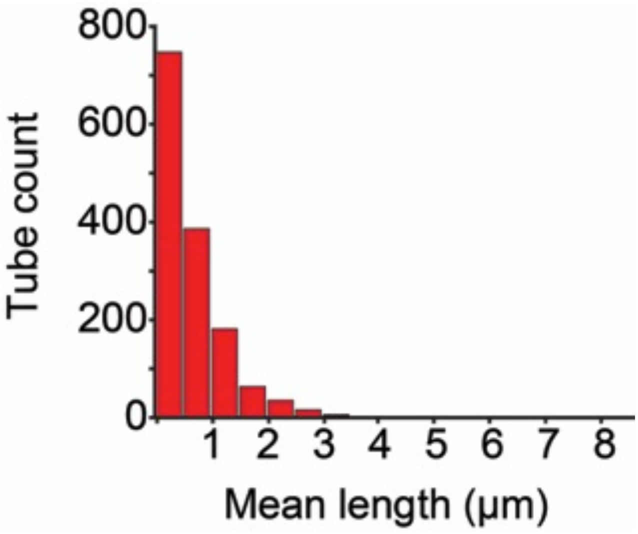

Supplementary Figure 19. Statistical analysis of tube mean lengths vs tube count obtained from the fluorescence microscopy images of Anti-Dig controlled nanotubes in the presence of Anti-Dig antibody (300 nM, see image of Figure 3c). See legend of Figure 3 for experimental details. Each bar in the histogram plot shows the total number of tubes observed over a 0.5 μm range of tube length.

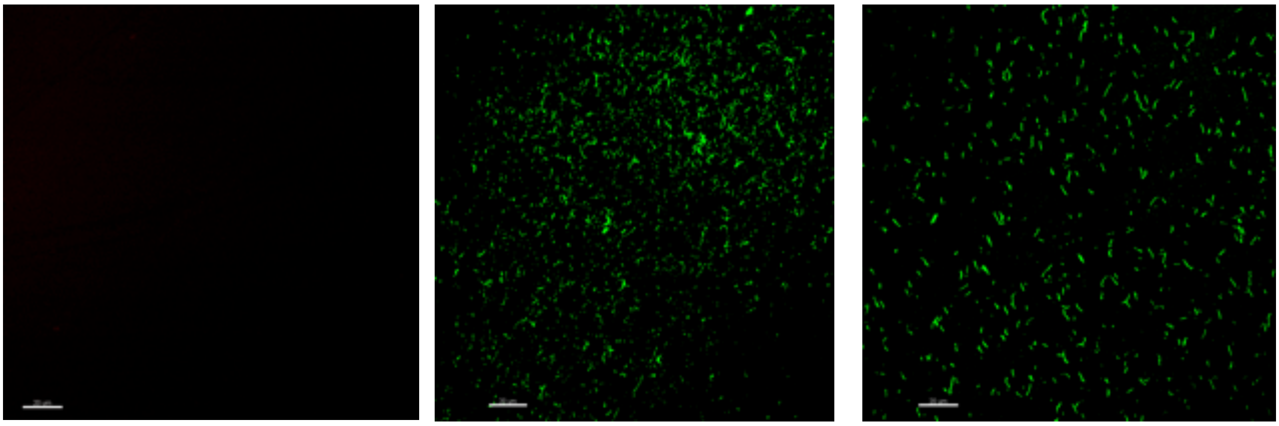

Supplementary Figure 20. Fluorescence microscopy images of nanotubes in the absence (left) and presence of Anti-DNP antibody (10 nM, center) (1 μM, right). See legend of Figure 3 for experimental details. Scale bars for these microscope images, 20 μm.

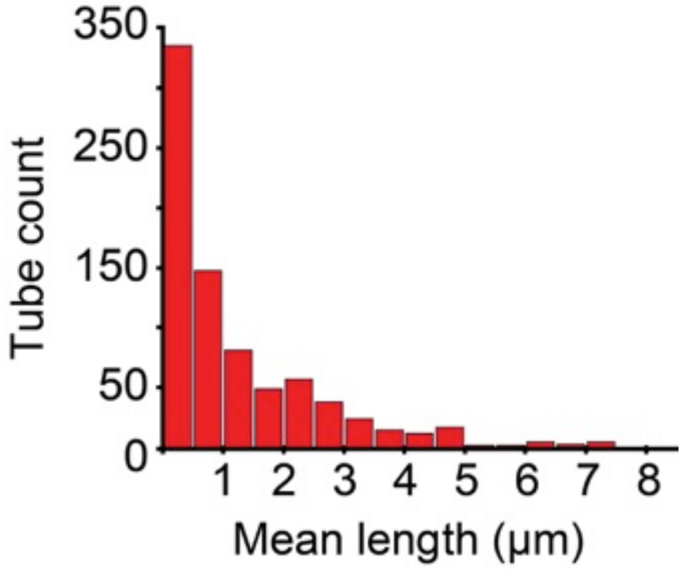

Supplementary Figure 21. Statistical analysis of tube mean lengths vs tube count obtained from the fluorescence microscopy images of Anti-DNP controlled nanotubes in the presence of Anti-DNP antibody (300 nM, see image of Figure 3f). See legend of Figure 3 for experimental details. Each bar in the histogram plot shows the total number of tubes observed over a 0.5 μm range of tube length.

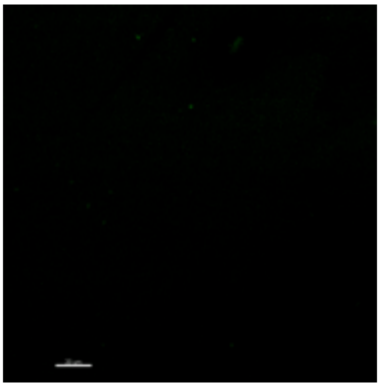

Supplementary Figure 22. Fluorescence microscopy images of nanotubes in the presence of Anti-Dig antibody (300 nM) using the anti-DNP controlled nanotube. No formation of DNA nanotubes is observed. See legend of Figure 3 for experimental details. Scale bars for these microscope images, 20 μm.

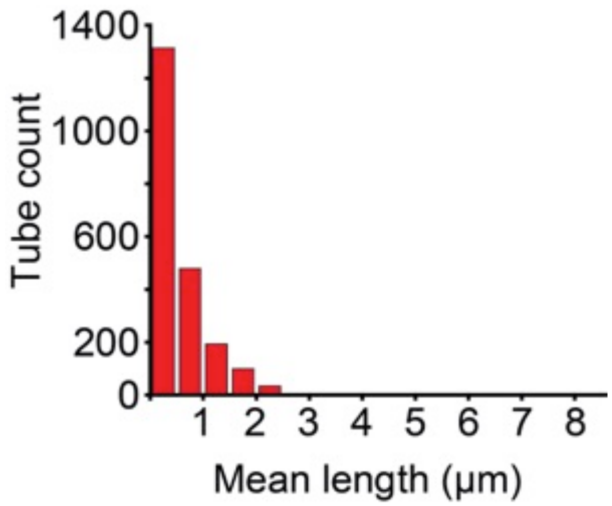

Supplementary Figure 23. Statistical analysis of tube mean lengths vs tube count obtained from the fluorescence microscopy images of Anti-Dig controlled nanotubes in the presence of Anti-Dig antibody (300 nM) for the orthogonal experiment (see Figure 3i, second from left). See legend of Figure 3 for experimental details. Each bar in the histogram plot shows the total number of tubes observed over a 0.5 μm range of tube length.

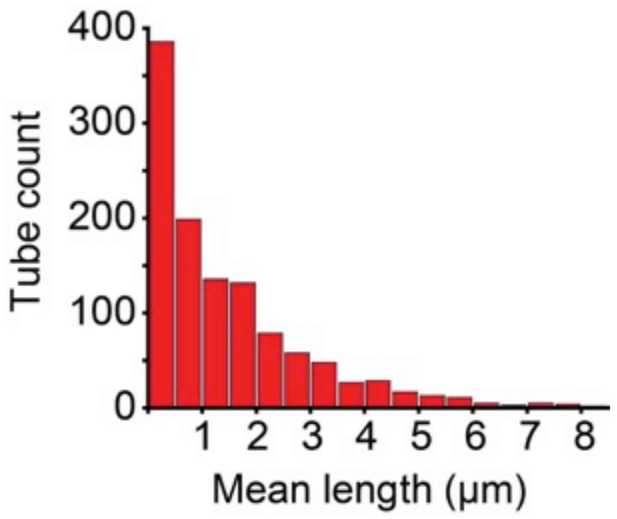

Supplementary Figure 24. Statistical analysis of tube mean lengths vs tube count obtained from the fluorescence microscopy images of Anti-DNP controlled nanotubes in the presence of Anti-DNP antibody (300 nM) for the orthogonal experiment (see Figure 3i, third from left). See legend of Figure 3 for experimental details. Each bar in the histogram plot shows the total number of tubes observed over a 0.5 μm range of tube length.

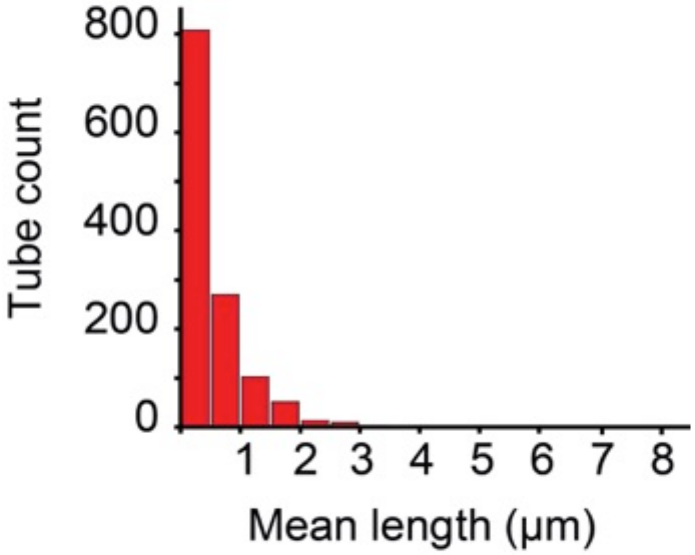

Supplementary Figure 25. Statistical analysis of tube mean lengths vs tube count obtained from the fluorescence microscopy images of Anti-Dig controlled nanotubes in the presence of Anti-Dig antibody (300 nM) and Anti-DNP antibody (300 nM) for the orthogonal experiment (see Figure 3i, right). See legend of Figure 3 for experimental details. Each bar in the histogram plot shows the total number of tubes observed over a 0.5 μm range of tube length.

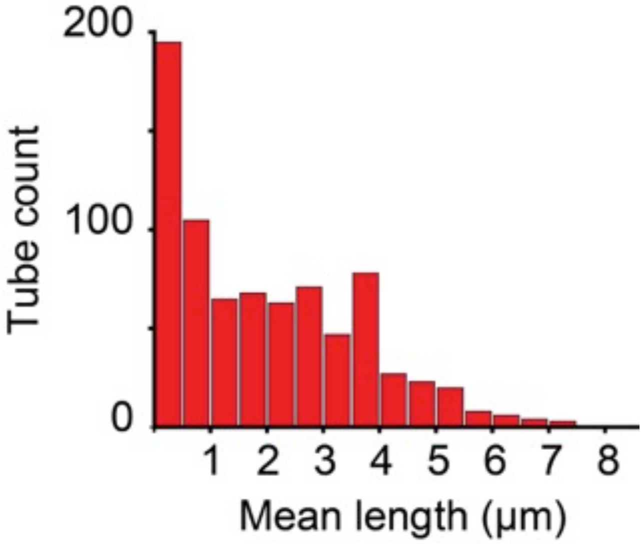

Supplementary Figure 26. Statistical analysis of tube mean lengths vs tube count obtained from the fluorescence microscopy images of Anti-DNP controlled nanotubes in the presence of Anti-Dig antibody (300 nM) and Anti-DNP antibody (300 nM) for the orthogonal experiment (see Figure 3i, right). See legend of Figure 3 for experimental details. Each bar in the histogram plot shows the total number of tubes observed over a 0.5 μm range of tube length.

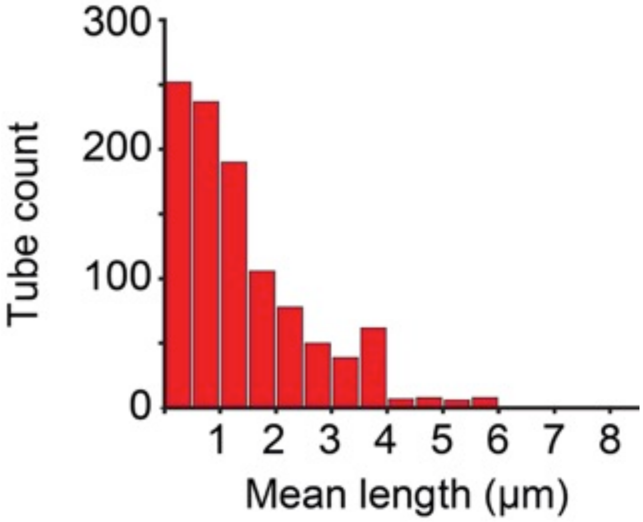

Supplementary Figure 27. Statistical analysis of tube mean lengths vs tube count obtained from the fluorescence microscopy images of Anti-DNP controlled nanotubes in the presence of Anti-DNP antibody (300 nM, see image of Figure 4b, center). See legend of Figure 4 for experimental details. Each bar in the histogram plot shows the total number of tubes observed over a 0.5 μm range of tube length.

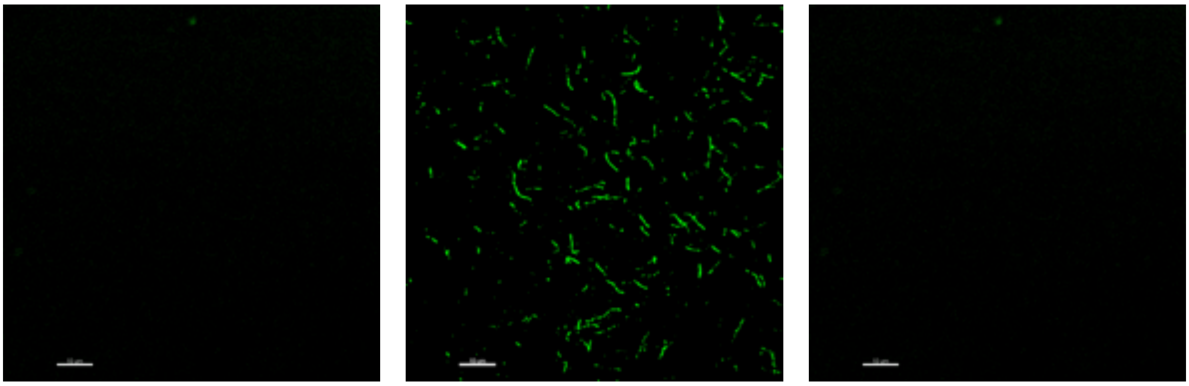

Supplementary Figure 28. Fluorescence microscopy images of nanotubes for the assembly/disassembly experiment (see Figure 4b) in the absence (left), presence of Anti-DNP antibody (300 nM, center) and Anti-DNP antibody + Anti- Dig antibody (both at 300 nM, right). See legend of Figure 4 for experimental details. Scale bars for these microscope images, 10 µm.

Supplementary References

1) Zadeh, J.N., Steenberg, C.D., Bois, J.S., Wolfe, B.R., Pierce, M.B., Khan, A.R., Dirks, R.M. & Pierce, N.A. Nupack: analysis and design of nucleic acid systems. *J. Comput. Chem.* **32**, 170–173 (2011).

2) Rothemund, P. W., Ekani-Nkodo, A., Papadakis, N., Kumar, A., Fygenson, D. K. & Winfree, E. Design and characterization of programmable DNA nanotubes. *J. Am. Chem. Soc.* **126**, 16344− 16352 (2004).
3) Mitchell, J. C., Harris, J. R., Malo, J., Bath, J. & Turberfield, A. J. Self-Assembly of Chiral DNA Nanotubes. *J. Am. Chem. Soc.* **126**, 16342−16343 (2004).
4) Zhang, D. Y., Hariadi, R. F., Choi, H. M. & Winfree, E. Integrating DNA strand-displacement circuitry with DNA tile self-assembly. Nat. Commun. 4, 1965 (2013).
5) Green, L. N., Amodio, A., Subramanian, H. K. K. S., Ricci, F. & Franco, E. pH-driven reversible self-assembly of micron-scale DNA scaffolds. *Nano Lett.* **17**, 7283–7288 (2017).
6) Green, L.N., Subramanian, H. K. K., Mardanlou, V., Kim, J. Hariadi, R.F. & Franco, E. Autonomous dynamic control of DNA nanostructure self-assembly. *Nat. Chem.* **11**, 510–520 (2019).
